# Ethnopharmacological Survey of Traditional Chinese Medicine Pharmacy Prescriptions for Dysmenorrhea

**DOI:** 10.3389/fphar.2021.746777

**Published:** 2021-12-21

**Authors:** Kuo-Han Su, Shan-Yu Su, Chien-Yu Ko, Yung-Chi Cheng, Shyh-Shyun Huang, Jung Chao

**Affiliations:** ^1^ Chinese Medicine Research Center, Department of Chinese Pharmaceutical Sciences and Chinese Medicine Resources, China Medical University, Taichung, Taiwan; ^2^ Department of Chinese Medicine, China Medical University Hospital, School of Post-Baccalaureate Chinese Medicine, College of Chinese Medicine, China Medical University, Taichung, Taiwan; ^3^ School of Pharmacy, China Medical University, Taichung, Taiwan; ^4^ Department of Pharmacology, Yale University School of Medicine, New Haven, CT, United States; ^5^ School of Pharmacy, China Medical University, Taichung, Taiwan; ^6^ Department of Food Nutrition and Health Biotechnology, Asia University, Taichung, Taiwan; ^7^ Master Program for Food and Drug Safety, Chinese Medicine Research Center, Department of Chinese Pharmaceutical Sciences and Chinese Medicine Resources, China Medical University, Taichung, Taiwan

**Keywords:** dysmenorrhea, ethnopharmacology, Taiwan, traditional Chinese medicine pharmacy, Chinese herbal medicines

## Abstract

Chinese herbal medicines have long been used for the treatment of dysmenorrhea. The treatment experiences of traditional Chinese medicine (TCM) pharmacies passed down through generations have contributed to a wealth of prescriptions for dysmenorrhea that have achieved significant therapeutic effects in countless Taiwanese women. Therefore, surveying and analyzing these prescriptions may enable us to elucidate the core medication combinations used in TCM prescriptions for dysmenorrhea. In the present study, a field investigation was conducted on various TCM pharmacies in Taiwan. A total of 96 TCM pharmacies were sampled, and 99 prescriptions for dysmenorrhea containing 77 different medicinal materials were collected. Compositae (8%) was the most common botanical source of the medicinal materials, and the predominant TCM property and flavor of the materials were warm (45%) and sweet (73%), respectively. The blood-activating and stasis-dispelling effect (23%) and the qi-tonifying effect (23%) were the most prevalent traditional effects, and the modern pharmacological effects most commonly found in the materials were anti-inflammatory (73%), antitumor (59%), and analgesic (12%) effects. Network analysis of the 77 medicinal materials used in the prescriptions, which was performed using the Traditional Chinese Medicine Inheritance Support System, yielded seven core medicinal materials and the corresponding network diagram. The seven core medicinal materials ranked in order of relative frequency of citation (RFC) were *Angelica sinensis* (Oliv.) Diels (Dang Gui), *Ligusticum chuanxiong* Hort (Chuan Qiong), *Rehmannia glutinosa* Libosch (Di Huang), *Paeonia lactiflora* Pall (Bai Shao), *Hedysarum polybotrys* Hand.-Mazz (Hong Qi), *Lycium chinense* Mill (Gou Qi Zi), and *Cinnamomum cassia* (L.). J. Presl (Gui Zhi). A total of 58 combinations, each consisting of two to five of the seven medicinal materials and 107 association rules among the materials, were identified. This study provides a record of valuable knowledge on TCM pharmacy prescriptions for dysmenorrhea. The rich medicinal knowledge of TCM pharmacies in Taiwan is worthy of further exploration, and the results of this study can serve as a basis for future pharmacological research and the development of naturally derived medications for dysmenorrhea.

## 1 Introduction

“Dysmenorrhea” is a Greek term meaning “difficult menstrual flow” (Burnett and Lemyre, 2017). Dysmenorrhea can be classified as primary and secondary dysmenorrhea, with the former defined as pain occurring with menses in the absence of pelvic pathology, and the latter as menstrual pain associated with underlying pelvic pathology, such as endometriosis (Burnett and Lemyre, 2017). It is a common condition among women of reproductive age, and the severe pain experienced by dysmenorrhea sufferers often causes interference with daily and educational activities (Durand et al., 2021). A review of relevant literature published between 1944 and 2015 revealed that primary dysmenorrhea affects 45–95% of menstruating women worldwide (Iacovides et al., 2015). Other studies have shown prevalence rates of 74–94% in European countries (Abreu-Sánchez et al., 2020; Barcikowska et al., 2020; Durand et al., 2021), 72.1% among young Asian women (Abubakar et al., 2020), and 65.3% in Taiwan (Yeh et al., 2019).

Treatment strategies for dysmenorrhea are broadly classified into three categories, namely pharmacological, surgical, as well as complementary and alternative therapies. Pharmacological therapy can be further divided into non-hormonal medical therapy, which includes various analgesics, and hormonal therapy, which includes combined hormonal contraceptives and progestin regimens (Burnett and Lemyre, 2017). This therapy is adopted by a large proportion of dysmenorrhea sufferers, with analgesics being used by up to 79.5% of affected women. However, pharmacological therapy produces side effects. For instance, non-steroidal anti-inflammatory drugs cause adverse effects in the gastrointestinal tract and central nervous system; hormonal treatment leads to side effects, such as nausea, breast tenderness, and headaches (Rosenberg et al., 1995), as well as an increased risk of venous thromboembolism (Vinogradova et al., 2014). Surgical management is indicated for secondary dysmenorrhea, and it involves open or laparoscopic surgery for eliminating pelvic pathology after a confirmed diagnosis has been made by pelvic ultrasound, magnetic resonance imaging (MRI), cystoscopy, or colonoscopy (Burnett and Lemyre, 2017). Complementary and alternative therapy is the most popular form of therapy among women with dysmenorrhea, with up to 95.1% of dysmenorrhea sufferers adopting non-pharmacological and non-surgical methods, such as heat application, hot shower/bath, exercise (Durand et al., 2021), transcutaneous electrical nerve stimulation, acupuncture and acupressure, behavioral interventions, and dietary supplements (Burnett and Lemyre, 2017).

In countries with widespread use of herbal medications, there have been reports of the adoption of herbs as a form of complementary and alternative therapy for dysmenorrhea. For instance, the use of *Marantodes pumilum* (family Primulaceae) to alleviate dysmenorrhea is popular in traditional Malay medicine (Aladdin et al., 2020). A study conducted in Turkey showed that the consumption of dry figs over three menstrual cycles decreased pain severity and increased quality of life (Amanak, 2020). Many ethnomedical studies have also shown that *Paeonia lactiflora* (Li et al., 2021), *Sparganium stoloniferum* (Jia et al., 2021), and *Foeniculum vulgare* (Lee et al., 2020) can alleviate pain in dysmenorrhea. Other studies have reported that *Tetradium ruticarpum* (Li and Wang, 2020) has been used to treat dysmenorrhea and pelvic inflammation in clinical practice for thousands of years, and that the fruit of *Akebia quinate* is widely used as a folk medicine to treat primary dysmenorrhea by the Tujia minority in China (Ma et al., 2021). Cinnamon, fennel, and ginger can effectively reduce pain intensity in primary dysmenorrhea, with cinnamon also being able to shorten the duration of pain (Xu Y. et al., 2020). A case-control study conducted in Ethiopia found that thyme tea drinking and consumption of vegetables and fruits have a primary dysmenorrhea-related pain-relieving tendency (Zeru and Muluneh, 2020).

In Taiwan, Chinese herbal medicines are the most popular form of complementary and alternative therapy used for treating dysmenorrhea, with approximately 75.2% of women aged 13–19 years and 63.3% of women aged 19–45 years in Taiwan seeking TCM treatment when suffering from dysmenorrhea (Huang, 2012). A previous survey showed that the majority of Taiwanese women purchased Chinese herbal medicines from community TCM pharmacies (Ho et al., 2011). However, as most of these TCM pharmacies are family-owned businesses, the Chinese herbal medicine knowledge of the pharmacists, including the processing methods, formulae, dosages, and administration methods, is only handed down by apprenticeship and has not been rigorously recorded or published (Huang et al., 2020). Hence, to gather and retain important medical knowledge regarding dysmenorrhea treatment that is currently only passed down from one generation to another in TCM pharmacies, we surveyed and gathered prescriptions for dysmenorrhea treatment from TCM pharmacies across Taiwan for the first time. Subsequently, the compositions of the prescriptions were analyzed to screen for the frequently used medicinal materials. The TCM property and flavor, traditional effects, and modern pharmacological effects of the frequently used medicinal materials were then subjected to statistical analysis and network analysis to determine the frequently used medicinal combinations and the core medicinal material network of the prescriptions. Our results may provide a scientific basis for future pharmacological research and the development of naturally derived medications for dysmenorrhea.

## 2 Materials and Methods

### 2.1 Ethical Review

The present study was conducted from October 2020 to April 2021 and was approved by the Central Regional Research Ethics Center of China Medical University prior to commencement (Approval No.: N/A/CRREC-109-125) (Supplementary Figure S1).

### 2.2 Research Process

**FIGURE 1:**
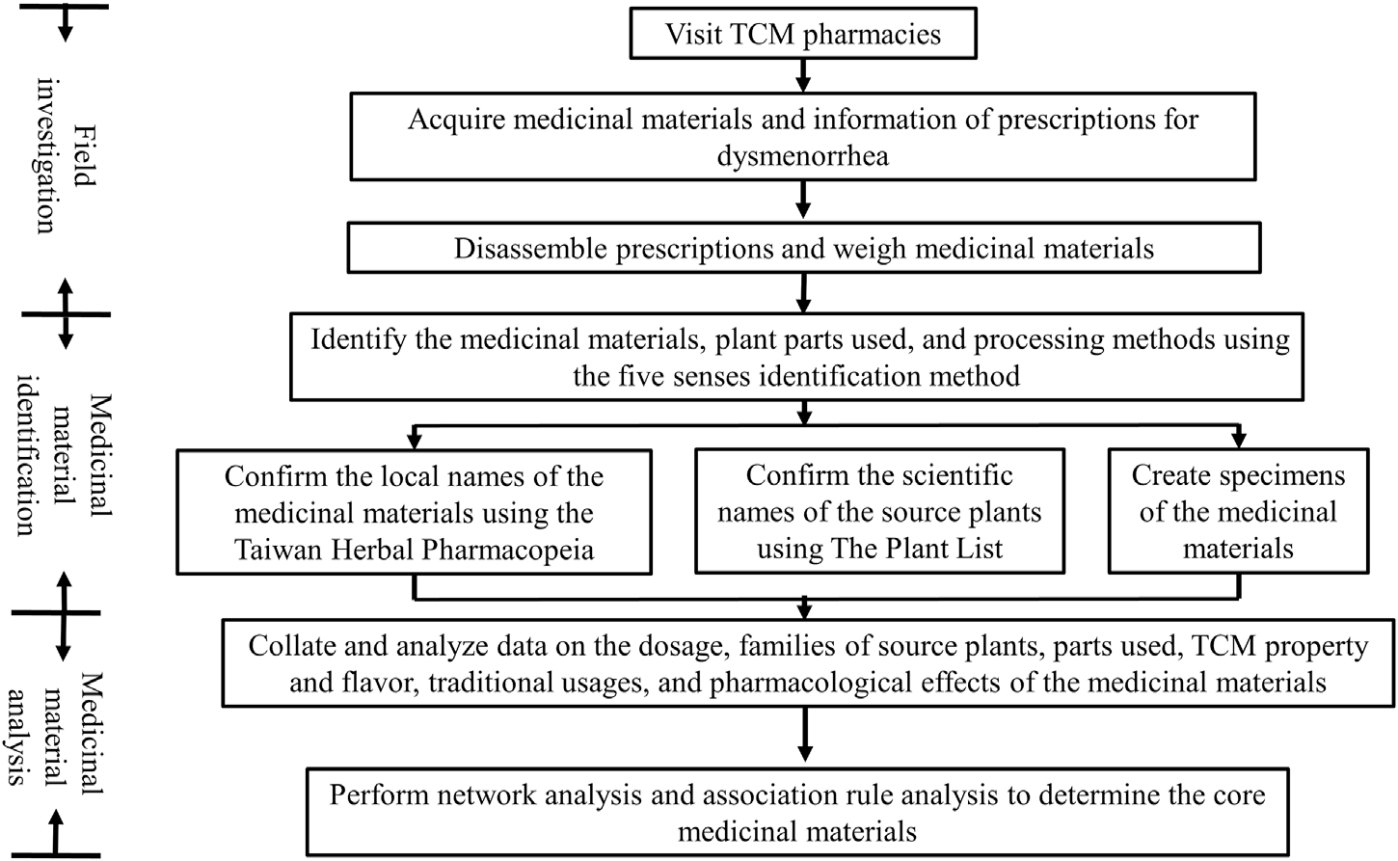
Research process flow chart.

The research process consisted of three main steps, namely field investigation, medicinal material identification, and medicinal material analysis (Figure 1).

### 2.3 Field Investigation

The field investigation of this study was performed on TCM pharmacies across the main island of Taiwan.

**FIGURE 2:**
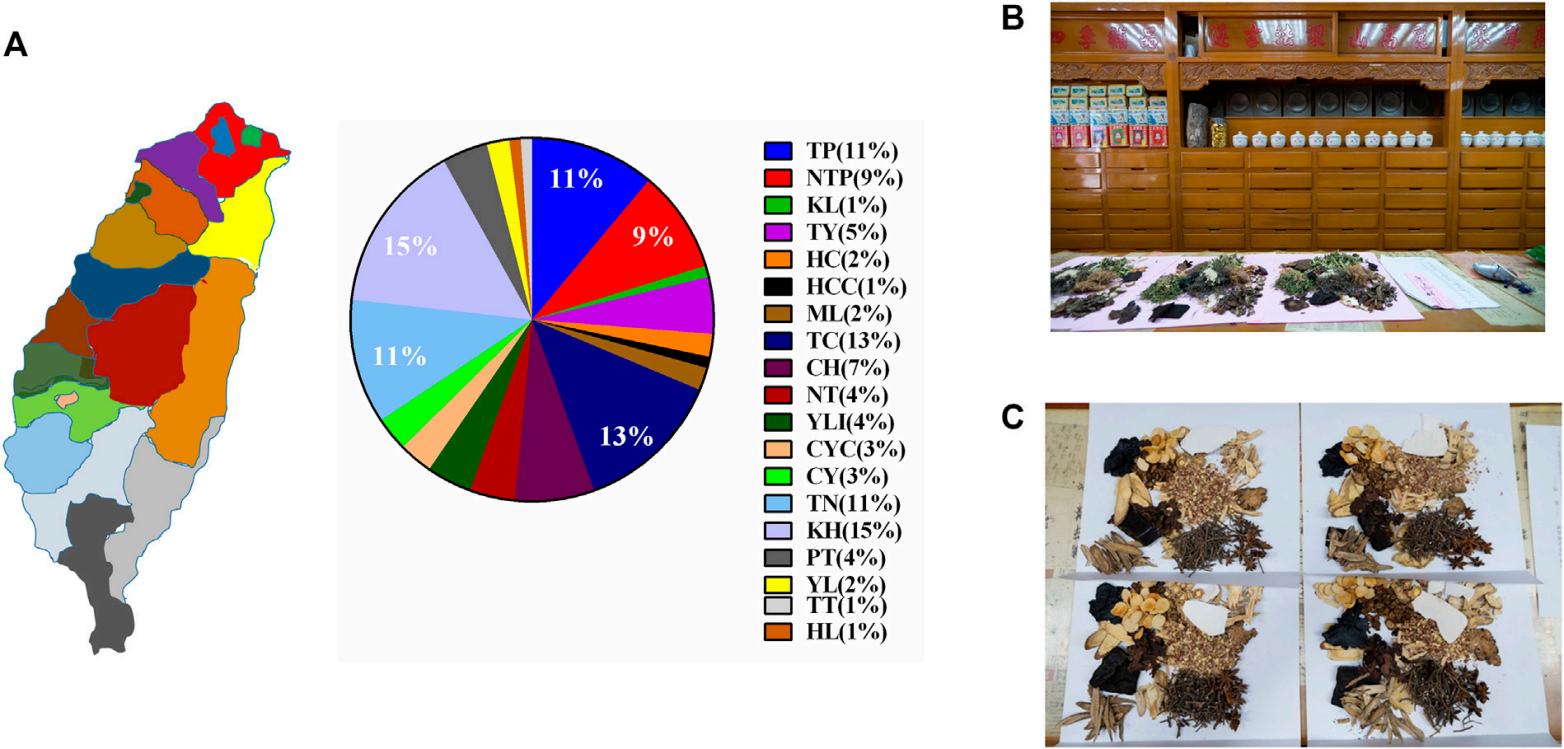
Regions investigated for prescriptions for dysmenorrhea (**A**) Distribution of TCM pharmacies across different regions in the main island of Taiwan; (**B**) Photograph of a typical TCM pharmacy in Taiwan; (**C**) Examples of TCM prescriptions collected during field investigation. CH, Changhua County; CYC, Chiayi City; CY, Chiayi County; HCC, Hsinchu City; HC, Hsinchu County; HL, Hualien County; KH, Kaohsiung City; KL, Keelung City; ML, Miaoli County; NT, Nantou County; NTP, New Taipei City; PT, Pingtung County; TC, Taichung City; TN, Tainan City; TP, Taipei City; TT, Taitung County; TY, Taoyuan City; YL, Yilan County, and YLI, Yunlin County.

The main island of Taiwan is located at 22–25°N, 120–122°E, and has a land area of approximately 36,000 km^2^. It measures approximately 395 km from north to south and has a maximum width of 144 km from east to west. The island country, located in the western side of the Pacific Ocean, has a combination of tropical and subtropical climates. Currently, the main island comprises six special municipalities, 10 counties, and three cities. TCM pharmacies across Taiwan were sampled using a ratio of 85:1 (96 pharmacies sampled from a total of 8,382 pharmacies) based on the proportions of registered pharmacy businesses in the various municipalities, counties, and cities (Figure 2A).

This study was conducted from October 2020 to April 2021. The research team visited TCM pharmacies across Taiwan for a field investigation, and purchased medicinal materials used in prescriptions for dysmenorrhea from select TCM pharmacies (Figure 2B).

### 2.4 Identification of Botanical Origin of Medicinal Materials

The purchased medicinal materials were disassembled (Figure 2C) for the identification of the origin, plant parts, and processing methods of the materials using the five senses identification method. We also photographed the materials and recorded the weight of each material. Finally, the materials were numbered and preserved in the herbarium of China Medical University. The taxonomic ranks and scientific names of all materials were determined in accordance with the taxonomy and nomenclature adopted in The Plant List.

### 2.5 Data Collation and Analysis

The following information of the medicinal materials used in the prescriptions for dysmenorrhea collected from TCM pharmacies across Taiwan was collated:

1) Names of medicinal materials: Scientific names and local names were determined using The Plant List and the third edition of the Taiwan Herbal Pharmacopeia (Ministry of Health and Welfare Taiwan, 2019), respectively.
2) TCM property and flavor, traditional usages, and frequently used doses: Data were obtained from the third edition of the Taiwan Herbal Pharmacopeia (Ministry of Health and Welfare Taiwan and Taiwan Herbal, 2019).
3) Relative frequency of citation (RFC): The frequency of citation (FC) of each material was first determined by summing the number of times that the material was used in the collected prescriptions. Subsequently, FC was divided by the total number of prescriptions collected in the study to obtain the RFC value (Chao et al., 2021), as shown by the following formula:

RFC = FC/the total number of prescriptions

4) Modern pharmacological effects: Relevant pharmacological studies published during the last 5 years were searched on PubMed (https://pubmed.ncbi.nlm.nih.gov/) using the scientific names of the medicinal materials as search terms.

### 2.6 Network Analysis of Associations of Medicinal Materials

Analysis of the associations among the medicinal materials was performed using the Traditional Chinese Medicine Inheritance Support System (TCMISS) V2.5, with support and confidence score set as 50% and 0.95, respectively. Support indicates the frequency with which a medicinal material appears in all collected prescriptions (Miao et al., 2019), and confidence score refers to the association of two materials among the various medicinal combinations, e.g., the confidence score is 0.95 if the probability of material B appearing when material A appears is 95% (Zucheng, 2020). The frequently used medicinal combinations and association rules obtained from association analysis were used to plot a network diagram of associations among the various materials, so as to determine the core medicinal materials used in the prescriptions for dysmenorrhea.

## 3 Results

### 3.1 Types and Taxonomic Characteristics of Medicinal Materials Used in Prescriptions for Dysmenorrhea Sold at TCM Pharmacies in Taiwan

A total of 99 prescriptions for dysmenorrhea were acquired from 96 TCM pharmacies during the field investigation. The prescriptions contained 77 different medicinal materials derived from organisms belonging to 45 families, with 73 materials derived from plants, 2 from fungi, and 2 from animals (Supplementary Table S1).

**FIGURE 3:**
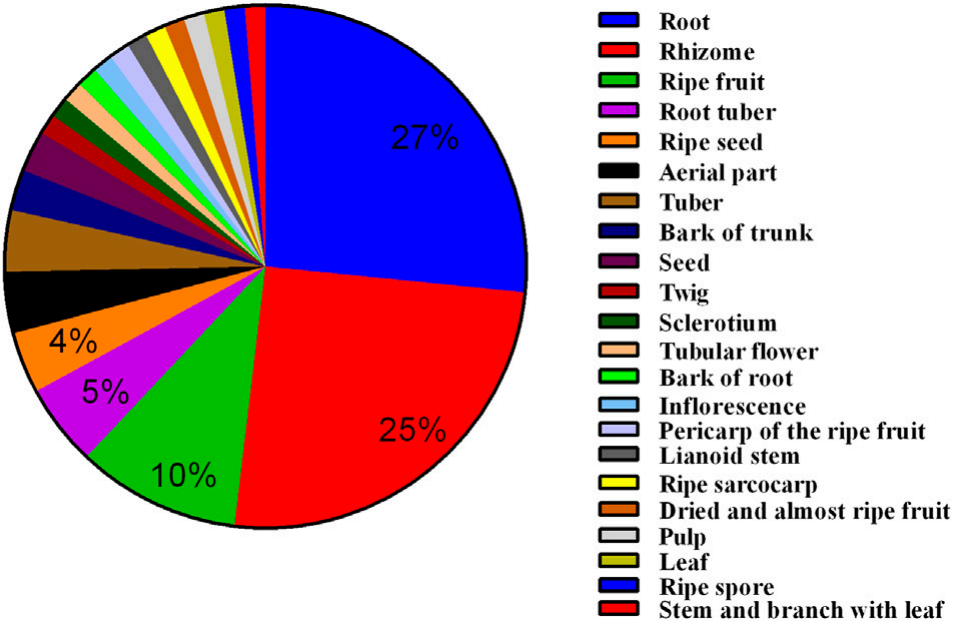
Utilized plant parts in the 77 medicinal materials used in prescriptions for dysmenorrhea.

An analysis of the plant parts used in the 77 medicinal materials revealed that the root was the most frequently utilized plant part (27%), followed by the rhizome (25%), ripe fruit (10%), root tuber (5%), ripe seed (4%), dried aerial part (4%), and tuber (4%) (Figure 3). All 77 medicinal materials were dried materials, which included the following processed materials: steamed Di Huang (*Rehmannia glutinosa* Libosch., abbreviated as RG), stir-baked Bai Shao (*Paeonia lactiflora* Pall., abbreviated as PL), stir-baked Du Zhong (*Eucommia ulmoides* Oliv., abbreviated as EU), honey-roasted Gan Cao (*Glycyrrhiza uralensis* Fisch., abbreviated as GU), and soil stir-baked Bai Zhu (*Atractylodes macrocephala* Koidz., abbreviated as AM).

**FIGURE 4:**
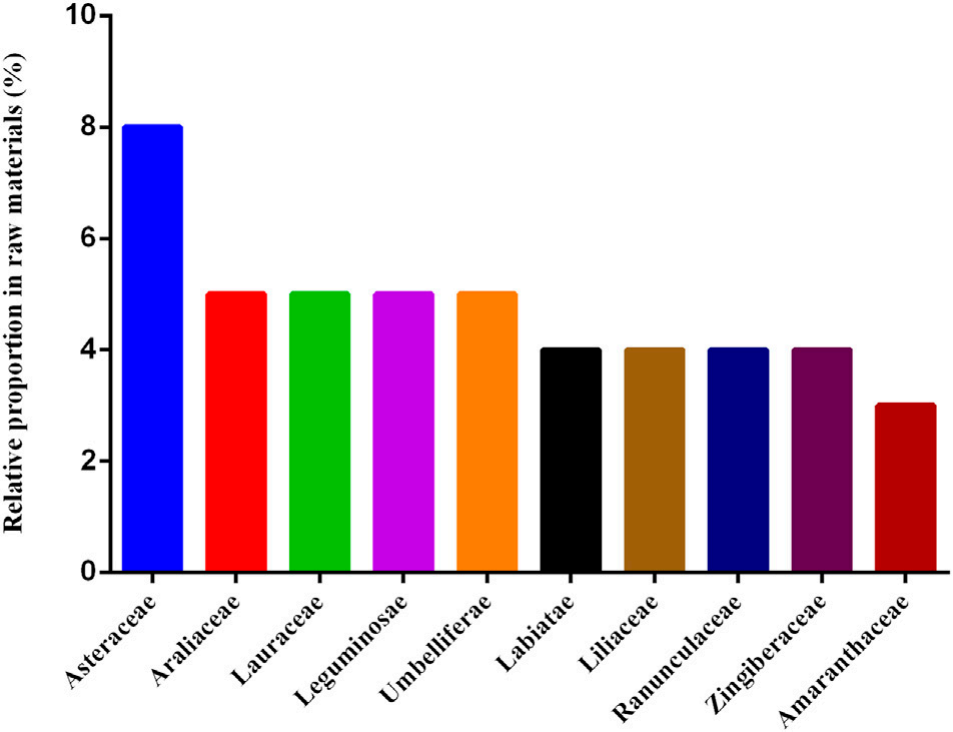
Top 10 families with the highest proportions in the 77 medicinal materials used in prescriptions for dysmenorrhea.

Members of the family Compositae accounted for the greatest proportion of the 77 medicinal materials (8%), followed by Araliaceae, Lauraceae, Leguminosae, and Umbelliferae (5% each), and Labiatae, Liliaceae, Ranunculaceae, and Zingiberaceae (4% each) (Figure 4).

### 3.2 Analysis of Frequently Used Medicinal Materials in Prescriptions for Dysmenorrhea

**FIGURE 5:**
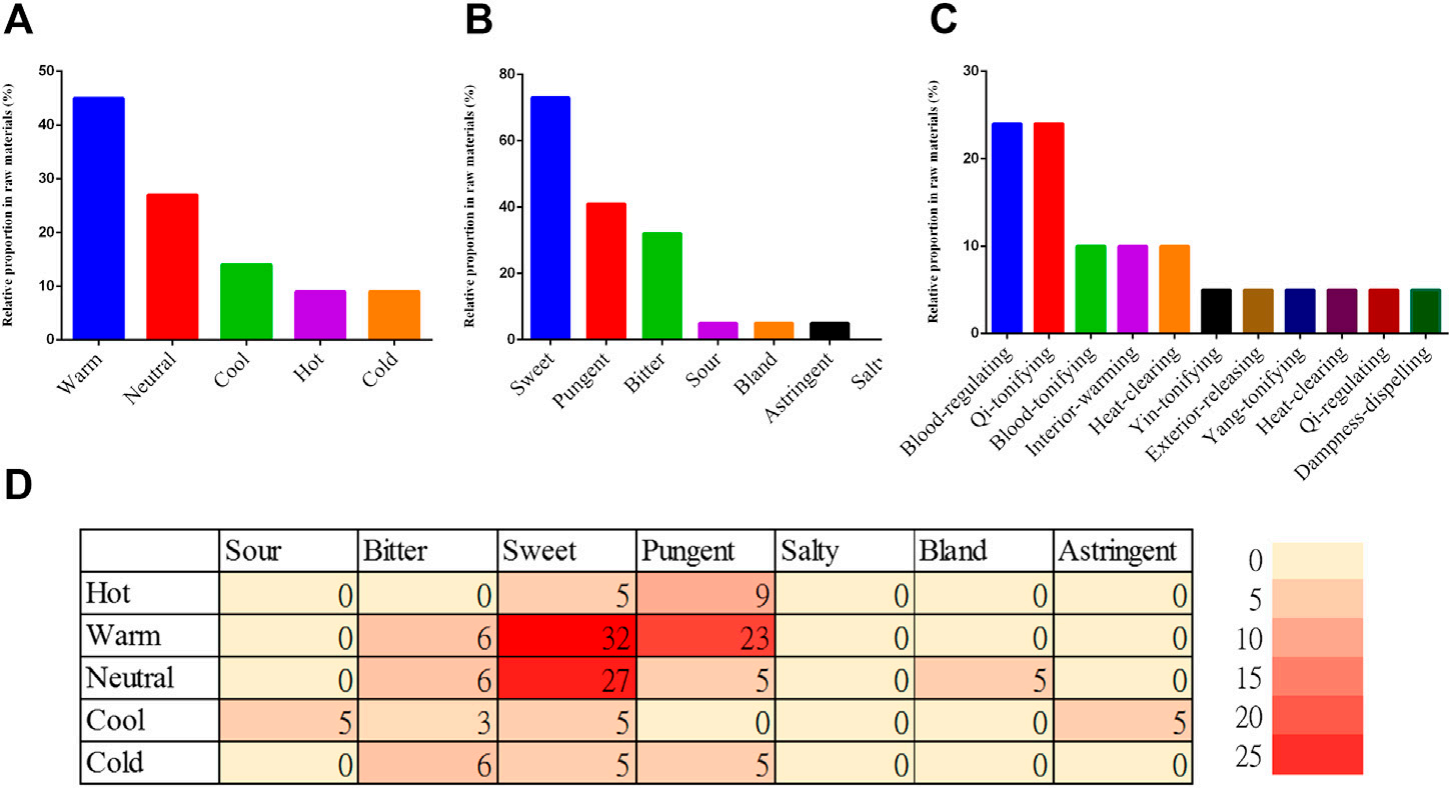
TCM property, flavor, and traditional effects of medicinal materials used in prescriptions for dysmenorrhea (**A**) TCM property; (**B**) TCM flavor; (**C**) Traditional effects; (**D**) Analysis of TCM flavor-property combinations; each number represents the proportion (%) of medicinal materials with the flavor-property combination among the 22 frequently used medicinal materials.

Among the 77 medicinal materials (Supplementary Table S1), 22 were frequently used in prescriptions for dysmenorrhea based on the criterion of RFC ≥0.1 (Table 1). The seven-most frequently used medicinal materials ranked in order of RFC were *Angelica sinensis* (Oliv.) Diels (Dang Gui, abbreviated as AS), *Ligusticum chuanxiong* Hort (Chuan Qiong, abbreviated as LiC), *Rehmannia glutinosa* Libosch. (Di Huang, abbreviated as RG), *Paeonia lactiflora* Pall. (Bai Shao, abbreviated as PL), *Hedysarum polybotrys* Hand.-Mazz (Hong Qi, abbreviated as HP), *Lycium chinense* Mill. (Gou Qi Zi, abbreviated as LyC), and *Cinnamomum cassia* (L.) J. Presl (Gui Zhi, abbreviated as CCT). The most common TCM flavor of the frequently used medicinal materials was sweet (73%), followed by pungent (41%). The most common TCM property was warm (45%), followed by neutral (27%). Among the various flavor-property combinations, sweet-warm was the most common (32%), followed by sweet-neutral (27%) and pungent-warm (23%) (Figure 5).

**TABLE 1:** Traditional effects and modern pharmacological effects of the 22 medicinal materials frequently used in prescriptions for dysmenorrhea (RFC ≥ 0.1).

No	Scientific name	Abbreviation	Family	Local name	Parts used	Dosage	Traditional usage	Property and flavor	Pharmacological effects	RFC
1	*Angelica sinensis* (Oliv.) Diels	AS	Umbelliferae	Dang Gui (當歸)	Roots	5–15 g	Tonifying and replenishing medicinal (blood-tonifying medicinal)	Warm; sweet and pungent	Antianemic and menstrual-pain-relieving effects (Li et al., 2015); anti-inflammatory and analgesic effects (Nie et al., 2009); antioxidant and anti-inflammatory effects (Yang et al., 2017; Cao et al., 2014)	0.96
2	*Ligusticum chuanxiong* Hort	LiC	Umbelliferae	Chuan Qiong (川芎)	Rhizome	3–10 g	Blood-regulating medicinal (blood-activating and stasis-dispelling medicine)	Warm; pungent	Anti-inflammatory and antioxidant effects (Shi et al., 2020)	0.95
3	*Rehmannia glutinosa* Libosch	RG	Scrophulariaceae	Di Huang (地黃)	Roots	9–30 g	Heat-clearing medicinal (heat-clearing and blood-cooling medicine)	Cold; sweet and bitter	Anti-inflammatory, antioxidant, and hypoglycemic effects. Kim et al. (2017)	0.79
4	*Paeonia lactiflora* Pall	PL	Ranunculaceae	Bai Shao (白芍)	Roots	6–15 g	Tonifying and replenishing medicinal (blood tonifying medicinal)	Mild cold; bitter and sour	Anti-inflammatory, antioxidant, antithrombotic, anticonvulsant, analgesic, cardioprotective, neuroprotective, hepatoprotective, antidepressant-like, antitumor, and immunoregulatory effects (Zhou et al., 2020)	0.71
5	*Hedysarum polybotrys* Hand.-Mazz	HP	Leguminosae	Hong Qi (紅耆)	Roots	9–30 g	Tonifying and replenishing medicinal (Qi tonifying medicinal)	Mild warm; sweet	Anti-gastric-ulcer effects (Yang et al., 2019)	0.68
6	*Lycium chinense* Mill	LyC	Solanaceae	Gou Qi Zi (枸杞子)	Ripe fruit	6–15 g	Tonifying and replenishing medicinal (Yin tonifying medicinal)	Neutral; sweet	Antioxidant, immunomodulatory, antitumor, neuroprotective, and hepatoprotective effects (Tian et al., 2019)	0.64
7	*Cinnamomum cassia* (L.) J.Presl	CCT	Lauraceae	Gui Zhi (桂枝)	Twig	3–10 g	Exterior-releasing medicinal (pungent-warm exterior-releasing medicinal)	Warm; pungent and sweet	Antitumor, anti-inflammatory, analgesic, antidiabetic, anti-obesity, antibacterial, antiviral, cardiovascular protective, cytoprotective, neuroprotective, immunoregulatory, and anti-tyrosinase activities Zhang et al. (2019)	0.59
8	*Glycyrrhiza uralensis* Fisch	GU	Leguminosae	Gan Cao (甘草)	Roots Rhizome	2–11.5 g	Tonifying and replenishing medicinal (Qi tonifying medicinal)	Neutral; sweet	Antiulcer, antimycobacterial, uterine relaxant, analgesic, antioxidant, memory-enhancing, corticosteroidal, antiallergic, hepatoprotective, anti-inflammatory, anticancer, antimalarial, antiviral, antihyperglycemic, antitussive, immunostimulatory, anti-HIV, muscle relaxant, and antimicrobial activities (Batiha et al., 2020)	0.45
9	*Codonopsis pilosula* (Franch.) Nannf.	CP	Campanulaceae	Dang Shen (黨參)	Roots	9–30 g	Tonifying and replenishing medicinal (Qi tonifying medicinal)	Neutral; sweet	Immunomodulatory, antitumor, antioxidant, neuroprotective, antiviral, anti-inflammatory, anti-fatigue, hypoglycemic, anti-hypoxia, renoprotective, gastroprotective, hepatoprotective, and prebiotic effects (Luan et al., 2021)	0.4
10	*Eucommia ulmoides* Oliv	EU	Eucommiaceae	Du Zhong (杜仲)	Bark of trunk	6–15 g	Tonifying and replenishing medicinal (Yang tonifying medicinal)	Warm; sweet	Antihypertensive, antihyperglycemic, antihyperlipidemic, antioxidant, anti-osteoporosis, antitumor, immunomodulatory, and neuroprotective activities (Wang et al., 2019)	0.38
11	*Ziziphus jujuba* Mill	ZJ	Rhamnaceae	Da Zao (紅棗)	Ripe fruit	6–30 g	Tonifying and replenishing medicinal (Qi tonifying medicinal)	Warm; sweet	Immunomodulatory, antioxidant, antitumor, hepatoprotective, and hypoglycemic activities, and gastrointestinal-protective effects. (Ji et al., 2017)	0.36
12	*Oroxylum indicum* (L.) Benth. ex Kurz	OI	Bignoniaceae	Mu Hu Dieh (木蝴蝶)	Seeds	1–4 g	Heat-clearing medicinal (heat-clearing and detoxicating medicinal)	Cool; bitter and sweet	Anticancer, antibacterial, hypoglycemic, cardioprotective, anti-adipogenesis, anti-inflammatory, and wound-healing effects (Nik Salleh et al., 2020)	0.31
13	*Ziziphus jujuba* Mill	ZJH	Rhamnaceae	Hei Zao (黑棗)	Ripe fruit	6–30 g	Tonifying and replenishing medicinal (Qi tonifying medicinal)	Warm; sweet	Immunomodulatory, antioxidant, antitumor, hepatoprotective, and hypoglycemic activities, and gastrointestinal-protective effects. (Ji et al., 2017)	0.3
14	*Cinnamomum cassia* (L.) J.Presl	CCB	Lauraceae	Rou Gui (肉桂)	Bark of trunk	1–5 g	Interior-warming medicinal	Highly hot; pungent and sweet	Antitumor, anti-inflammatory, analgesic, antidiabetic, anti-obesity, antibacterial, antiviral, cardiovascular protective, cytoprotective, neuroprotective, immunoregulatory, and anti-tyrosinase effects Zhang et al. (2019)	0.28
15	*Poria cocos* (Schwein.) F.A.Wolf	PC	Polyporaceae	Fu Ling (茯苓)	Sclerotium	9–30 g	Dampness-dispelling medicinal (dampness-draining, diuretic medicinal)	Neutral; sweet and bland	Antitumor, immunomodulatory, anti-inflammatory, antioxidant, antiaging, anti-hepatitis, antidiabetic, and anti-hemorrhagic-fever effects Li et al. (2019)	0.27
16	*Atractylodes macrocephala* Koidz	AM	Compositae	Bai Zhu (白朮)	Rhizome	6–15 g	Tonifying and replenishing medicinal (Qi tonifying medicinal)	Warm; bitter and sweet	Antitumor, neuroprotective, anti-hepatotoxicity, and anti-inflammatory effects (Ruqiao et al., 2020)	0.25
17	*Prunus persica* (L.) Batsch	PP	Rosaceae	Tao Ren (桃仁)	Ripe seed	4.5–10 g	Blood-regulating medicinal (blood-activating and stasis-dispelling medicinal)	Neutral; bitter and sweet	Anti-obesity effect (Song et al., 2019); anti-inflammatory, antinociceptive, and antipyretic effects (Elshamy et al., 2019)	0.21
18	*Zingiber officinale* Roscoe	ZO	Zingiberaceae	Gan Jiang (乾薑)	Rhizome	3–9 g	Interior-warming medicinal	Hot; pungent	Antiemetic, antibacterial, antitumor, anti-inflammatory, and antioxidant effects Li et al. (2021)	0.17
19	*Cyperus rotundus* L	CR	Cyperaceae	Xiang Fu (香附)	Rhizome	6–11.5 g	Qi-regulating medicinal	Neutral; pungent, mild bitter and mild sweet.	Analgesic, anti-allergic, anti-arthritic, anti-candida, anticariogenic, anticonvulsant, antidiarrheal, antiemetic, antihelminthic, antihistamine, antihyperglycemic, antihypertensive, anti-inflammatory, antimalarial, anti-obesity, antioxidant, antiplatelet, antipyretic, anti-ulcer, antiviral, cardioprotective, cytoprotective, cytotoxic, gastroprotective, hepatoprotective, neuroprotective, ovicidal, larvicidal, and wound healing effects as well as inhibitory effect on Na^+^ K^+^ ATPase activities in the brain (Kamala et al., 2018)	0.15
20	*Leonurus japonicus* Houtt	LJ	Labiatae	Yi Mu Cao (益母草)	Aerial part	9–30 g	Blood-regulating medicinal (blood-activating and stasis-dispelling medicinal)	Mild cold; bitter and pungent	Antioxidant, anti-inflammatory, and antiapoptotic effects (Huang et al., 2021)	0.14
21	*Carthamus tinctorius* L	CaT	Compositae	Hong Hua (紅花)	Tubular flower	3–10 g	Blood-regulating medicinal (blood-activating and stasis-dispelling medicinal)	Warm; pungent	Cardioprotective, neuroprotective, anticancer, and anticoagulant effects (Orgah et al., 2020)	0.1
22	*Corydalis yanhusuo* W.T. Wang	CY	Papaveraceae	Yan Hu Su (延胡索)	Tuber	3–12 g	Blood-regulating medicinal (blood-activating and stasis-dispelling medicinal)	Warm; pungent and bitter	Antianxiety, hypnosis-inducing effect, analgesic, anti-arrhythmic, anti-ulcer, and anti-myocardial-ischemia effects (Tian et al., 2020)	0.1

The most prevalent traditional effects of the frequently used medicinal materials were the blood-activating and statis-dispelling effect (23%) and the qi-tonifying effect (23%), followed by the blood-tonifying effect, interior-warming effect, and heat-clearing and blood-cooling effect (9% each) (Figure 5).

**FIGURE 6:**
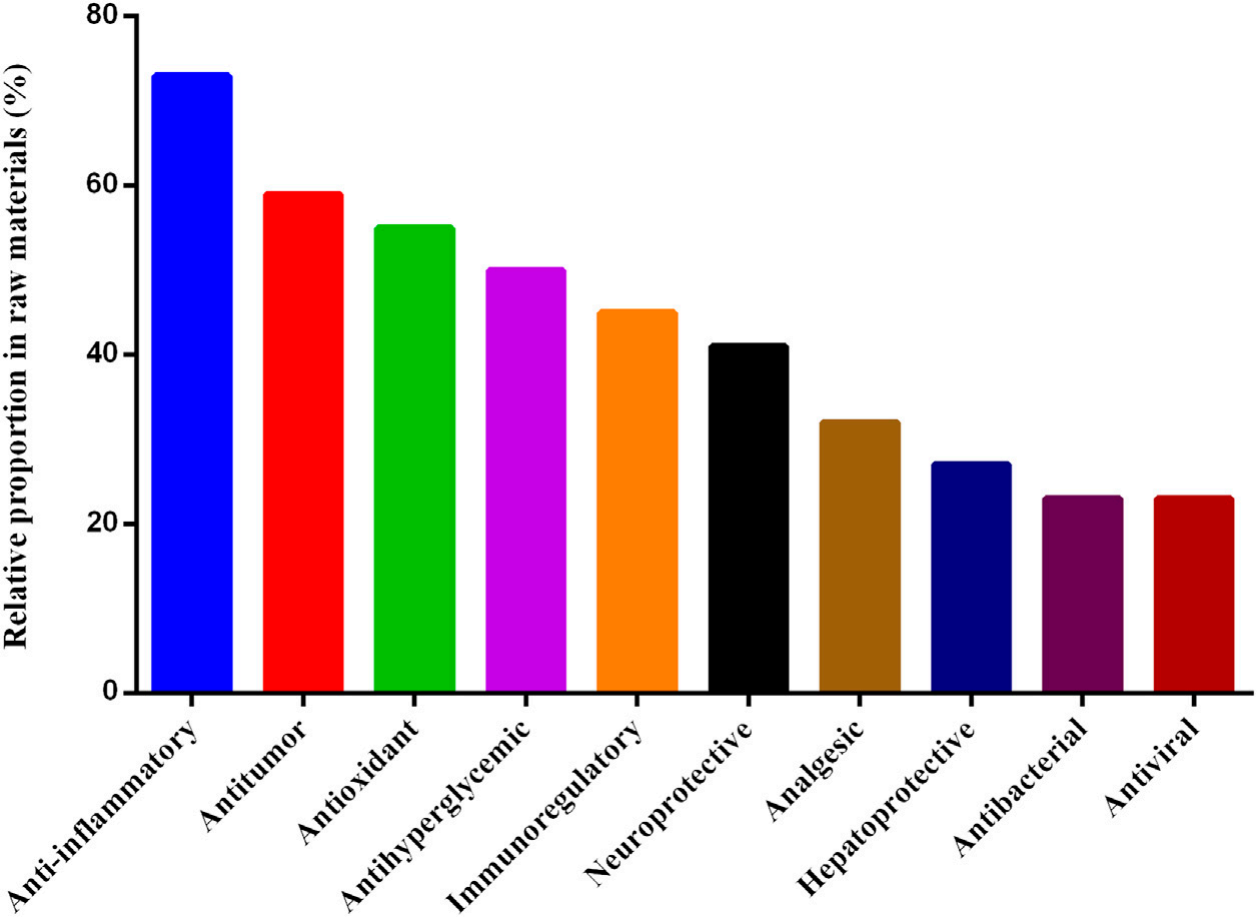
Modern pharmacological effects of the frequently used medicinal materials.

As shown in Figure 6, the most commonly reported modern pharmacological effect of the frequently used medicinal materials was anti-inflammatory effect, which was reported for 16 of the 22 frequently used medicinal materials (73%). This was followed by antitumor (59%), antioxidant (20%), Antihyperglycemic (19%), immunomodulatory (17%), neuroprotective (15%), analgesic (12%), hepatoprotective (10%), antibacterial (8%), and antiviral (8%) effects (Figure 6).

**FIGURE 7:**
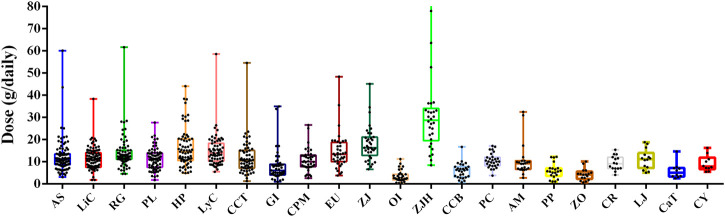
Box plot of dose ranges of frequently used medicinal materials (top line denotes the maximum value, and bottom line denotes the minimum value; the top edge of each box represents the third quartile (Q3), middle line represents the second quartile (Q2), and bottom edge represents the first quartile (Q1). Black dots represent the doses in the collected samples.; AS, *Angelica sinensis* (Oliv.) Diels; AM, *Atractylodes macrocephala* Koidz.; CCT, *Cinnamomum cassia* (L.) J. Presl; CPM, *Codonopsis pilosula* (Franch.) Nannf. var. *modesta* (Nannf.) L.T.Shen; CCB, *Cinnamomum cassia* (L.) J. Presl; CR, *Cyperus rotundus* L.; CaT, *Carthamus tinctorius* L.; CY, *Corydalis yanhusuo* W.T.Wang.; DL, *Diospyros lotus*; EU, *Eucommia ulmoides* Oliv.; GI, *Glycyrrhiza inflata* Batalin; HP, *Hedysarum polybotrys* Hand.-Mazz.; LiC, *Ligusticum chuanxiong* Hort; LyC, *Lycium chinense* Mill.; LJ, *Leonurus japonicus* Houtt.; OI, *Oroxylum indicum* (L.) Benth. ex Kurz; PL, *Paeonia lactiflora* Pall.; PC, *Poria cocos* (Schwein.) F.A.Wolf; PP, *Prunus persica* (L.) Batsch; RG, *Rehmannia glutinosa* Libosch.; ZJ, *Ziziphus jujuba* Mill.; ZO, *Zingiber officinale* Roscoe.

Among the various medicinal materials used in the prescriptions, *Diospyros lotus* (Hei Zao, abbreviated as ZJH) had the highest average dose of 25.18 ± 8.48 g (Supplementary Table S2), and *Oroxylum indicum* (L.) Benth. ex Kurz (Mu Hu Die, abbreviated as OI) had the lowest average dose of 3.09 ± 2.04 g. DL (25.18 ± 8.48 g) and HP (15.12 ± 7.24 g) showed the largest dose differences across the various TCM pharmacies, whereas *Poria cocos* (Schwein.) F.A.Wolf (Fu Ling, abbreviated as PC), *Prunus persica* (L.) Batsch (Tao Ren, abbreviated as PP), *Zingiber officinale* Roscoe (Gan Jiang, abbreviated as ZO), and *Cyperus rotundus* L. (Xiang Fu, abbreviated as CR) exhibited the smallest dose differences across various TCM pharmacies (Figure 7).

### 3.3 Association and Network Analysis of Medicinal Materials Used in Prescriptions for Dysmenorrhea

Analysis of the associations among the 77 medicinal materials used in prescriptions for dysmenorrhea was performed using TCMISS with the support and confidence scores set as >50% and >0.95, respectively. A total of 58 frequently used combinations were obtained (Supplementary Table S3), with 19 being two-material combinations, 22 being three-material combinations, 13 being four-material combinations, and three being five-material combinations.

**FIGURE 8:**
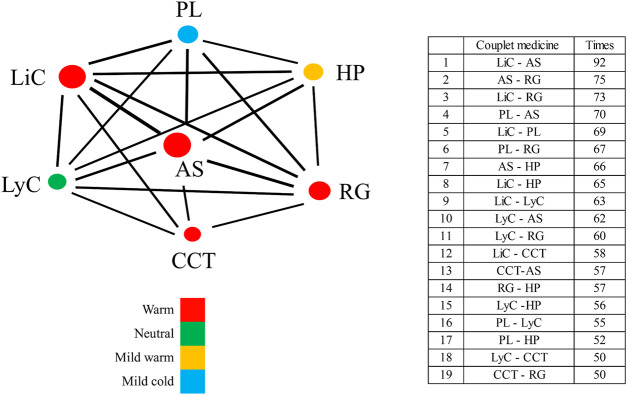
Network diagram of core medicinal materials used in prescriptions for dysmenorrhea. Different colors represent different TCM properties. Circle sizes are proportional to the respective FC values, and line thicknesses are proportional to the respective frequencies of two-material combinations. AS, *Angelica sinensis* (Oliv.) Diels; CCT, *Cinnamomum cassia* (L.) J. Presl; HP, *Hedysarum polybotrys* Hand.-Mazz.; LiC, *Ligusticum chuanxiong* Hort; LyC, *Lycium chinense* Mill.; PL, *Paeonia lactiflora* Pall.; RG, *Rehmannia glutinosa* Libosch.

Network analysis performed on the associations of these medicinal combinations revealed a total of seven core medicinal materials used in the prescriptions for dysmenorrhea (Figure 8). The core materials ranked in order of FC were AS, LiC, RG, PL, HP, LyC, and CCT (Figure 8). Two-core material combinations with the highest FC were LiC-AS (92), AS-RG (75), and LiC-RG (73); three-core material combinations with the highest FC were LiC-AS-RG (73), LiC-PL-AS (69), and PL-AS-RG (67); four-core material combinations with the highest FC were LiC-PL-AS-RG (66), LiC-LyC-AS-RG (60), and LiC-AS-RG-HP (56). The five-core material combination with the highest FC was LiC-PL-LyC-AS-RG (55), i.e., 55 of the 99 acquired prescriptions for dysmenorrhea contained these five core medicinal materials.

Results of association rule analysis showed the presence of 107 association rules (Supplementary Table S4). Two-material associations with a confidence value of 1 included CCT → LiC, LyC → LiC, PL→AS, and RG → AS. In other words, when support >50%, the probability that the medicinal material on the right side of the arrow appeared in the same prescription as the medicinal material on the left side of the arrow was 100%.

## 4 Discussion

### 4.1 Ethnopharmacological Investigation Sites

The remaining TCM pharmacies in Taiwan are an invaluable asset to ethnopharmacology because a considerable amount of knowledge regarding Chinese herbal medicines, TCM formulations, and medicinal material processing techniques is contained exclusively within these age-old pharmacies. However, such pharmacies have gradually become a sunset industry with the gradual westernization of the Taiwanese society (Kuo, 2021). This has resulted in an urgent need for the systemic retention and documentation of valuable ethnopharmacological knowledge (Chao et al., 2021). Thus, in this study, we surveyed and collated prescriptions for dysmenorrhea treatment from TCM pharmacies across Taiwan.

### 4.2 Taxonomic Characteristics of Medicinal Materials Used in Prescriptions for Dysmenorrhea

Members of the Asteraceae (Compositae) family accounted for the greatest proportion of the medicinal materials used for dysmenorrhea. This finding echoes a previous study that concluded that plants belonging to the Compositae family constitute the major source of TCM herbs in Taiwan (Huang et al., 2020). The properties and the flavors of Compositae are often cool and bitter, and there are not many medicinal materials of Compositae family are warm (Wei, 2014). However, the present investigation found that medicinal materials of Compositae used in dysmenorrhea are mostly warm, such as AM, *Carthamus tinctorius* L. (Hong Hua), *Artemisia argyi* H. Lév. and Vaniot (Ai Ye), etc. Compositae plants used in dysmenorrhea are very different from those used in other diseases. The family Leguminosae is also a main source of medicinal materials frequently used in prescriptions for dysmenorrhea, which include *Astragalus membranaceus* (Fisch.) Bge (Huang Qi), HP, and GU. Leguminosae plants have also been used in the treatment of infertility and menopausal complaints in Cameroon (Ateba et al., 2013) and as galactagogues in Taiwan (Chao et al., 2021). Other plant families frequently used as medicinal materials in prescriptions for dysmenorrhea include Araliaceae (e.g., *Panax ginseng* and *Panax quinquefolius*), Lauraceae (e.g., *Cinnamomum cassia* (L.) J. Presl (Rou Gui, abbreviated as CCB) and CCT), and Umbelliferae (e.g., AS, LiC, and *Radix Bupleuri* (Chai Hu)).

### 4.3 Traditional Effects and Modern Pharmacological Effects of Medicinal Materials Frequently Used in Prescriptions for Dysmenorrhea

#### 4.3.1 TCM Properties, Flavors, and Traditional Effects of Prescriptions for Dysmenorrhea

Traditional Chinese medicine has a unique theoretical framework, including four characteristics, five flavors and so on. Cold, hot, warm, cool and neutral represent different medicinal properties (Guan et al., 2009). The ^1^H-NMR spectrum results show that there are obvious differences in the chemical composition of Chinese medicines with different medicinal properties (Zhang et al., 2020). Moreover, “cold” nature-related drugs have more fatty rings, while “hot” nature-related drugs have lower average molecular weight and more aromatic ring systems. “Neutral” nature-related drugs have more cyclohexene (Fu et al., 2017).

Among the various flavor-property combinations, sweet-warm, sweet-neutral, and pungent-warm were the most common in the prescriptions for dysmenorrhea. In TCM theory, sweet medicinal materials are regarded as having tonifying and replenishing effects and being capable of relaxing tension and relieving pain; warm and hot materials are used for the treatment of cold-type diseases, such as aversion to cold; pungent materials can promote qi and blood flow, and are used to treat symptoms of poor blood circulation (Wei, 2019). Warm and hot materials are also related to enhance fertility, sexual function, and endocrine, nutrition and metabolic state (Fu et al., 2017).

The most prevalent traditional effects of the frequently used medicinal materials were the blood-activating and statis-dispelling effect as well as the qi-tonifying effect, which were each reported in five materials. This was followed by the blood-tonifying effect, interior-warming effect, and heat-clearing and blood-cooling effect (9% each) (Table 1). The most common TCM syndrome type of primary dysmenorrhea is Qi stagnation with blood stasis syndrome, indicating the interruption of blood flow would cause menstrual pain (Park et al., 2012; Kim et al., 2017). The high prevalence of the blood-activating, blood-tonifying, qi-tonifying, and interior-warming effects among the frequently used medicinal materials is consistent with the principles of dysmenorrhea treatment in TCM (Xin, 2021).

#### 4.3.2 Modern Pharmacology and Dysmenorrhea

Among the seven core medicinal materials that are frequently used for dysmenorrhea (Figure 8), AS showed the highest RFC among the prescriptions. It is also an edible traditional medicinal plant in China, Japan, and South Korea. The organic acids contained in AS has been shown to reduce inflammation by enhancing the autophagy flux of damaged endothelial cells (Li et al., 2020). In clinical practice, AS is used for blood replenishment and the treatment of irregular menstruation and dysmenorrhea (Wei et al., 2016). Ferulic acid, a component of AS, reduces the secretion of expression of interleukin-8 and vascular endothelial growth factor in endometriotic tissues, which explains the therapeutic effect of dysmenorrhea (Takeuchi et al., 2020). LiC has been widely used in the treatment of thrombotic diseases and can reduce the levels of pro-inflammatory cytokines (IL-1β, TNF-α) (Wang et al., 2020). Research has shown that the main active constituents of PL are monoterpene glucosides, which possess antioxidant and anti-inflammatory effects (Li et al., 2021). In particular, paeoniflorin, which is one of the monoterpene glucosides and a major active compound of PL, improves endometrial receptivity by inducing the expression of leukemia inhibitory factors, thereby enhancing the embryo implantation rate (Park et al., 2021). PL may also provide beneficial effects towards ovarian function and oocyte quality, possibly by stimulating ovarian angiogenesis and follicular development (Park et al., 2020). Consequently, PL has been used as an important herbal remedy for the treatment of dysmenorrhea in many ethnomedical medical systems around the world (Li et al., 2021). The main constituents of CCT are terpenoids, phenylpropanoids, and glycosides, and modern studies have confirmed that CCT possesses a wide range of pharmacological effects, including anti-inflammatory and analgesic effects (Zhang et al., 2019). *Trans*-cinnamaldehyde, a bioactive component found in CCT, exhibited good anti-inflammatory effects in a lipopolysaccharide-induced zebrafish inflammation model and rat experiments (Lee and Lim, 2021; Park et al., 2021). *Lycium barbarum* polysaccharides, which are the active component of LyC, exerted protective effects against ovarian injury in rats by reducing oxidative stress and activating the Nrf2/ARE-signaling pathway (Yang et al., 2017). Therefore, the treatment of dysmenorrhea by the aforementioned medicinal materials may be related to their anti-inflammatory, antioxidant, and analgesic effects. Further research will be required for the validation of this conjecture.

### 4.4 Combinations, Doses, and Processing of Medicinal Materials Used in Prescriptions for Dysmenorrhea

The seven core medicinal materials result from network analysis includes warm (AS, LiC, RH, CCT, and HP), neutral (LyC), and cool herbs (PL). The four materials with highest RFC, including LiC, AS, RG, and PL, is exactly a traditional formula, the Si Wu (four-substance) Decoction (Dan 2020). Association analysis showed that the four materials are the most frequently combined materials in the prescriptions. The daily doses of the four medicinal materials in the prescriptions for dysmenorrhea were almost the same, i.e. 11 g per day. This gives a dose ratio of close to 1:1:1:1, which is almost identical to the dose ratio used in the formula for the Si Wu decoction in the original use (Lai et al., 2020). In a study by Li et al., a network of “compound-target-pathway-disease” of the Si Wu Decoction was constructed, and network analysis showed that 16 components, 16 target proteins, and 24 pathways of the decoction were related to primary dysmenorrhea (Li et al., 2019). The four medicinal materials may play a role in treating dysmenorrhea by acting on protein targets and pathways related to hormone regulation, analgesia, spasmolysis, inflammation, and immunity (Li et al., 2019). In the present study, the fifth-most frequently used core medicinal material used in the prescriptions for dysmenorrhea was HP, which has long been used as an alternative to *Astragalus membranaceus* (Fisch.) Bge (Huang Qi) in Taiwan (Chao et al., 2020). LyC and CCT, with respective RFC values of 0.64 and 0.59, were also frequently used in prescriptions for dysmenorrhea.

The purpose of medicinal materials processing is to enhance the therapeutic efficacy and reduce the toxicity of original medicinal materials, by using vinegar, wine, honey, brine and other auxiliary materials. Scientific reports show that processing has a synergistic effect on the chemistry, pharmacology and pharmacokinetics with the active ingredients of medicinal materials (Chen et al., 2018). Processed medicinal materials in the prescriptions for dysmenorrhea included steamed RG, stir-baked PL, stir-baked EU, honey-roasted GU, and soil stir-baked AM. Steaming increases the anti-inflammatory and hematopoietic effects of RG, which significantly improves hematopoiesis in the body after consumption (Wang et al., 2018). The extracts of stir-baked PL promote the synthesis and release of IL-4 and IL-10 and inhibit the expression of IL-1β, TNF-α, and high mobility group box 1 protein (HMGB1), thereby providing anti-inflammatory and analgesic effects (Xian-wen, 2020). It has been found that the alcohol extract of stir-baked EU has a significantly higher EU content than the alcohol extract of raw EU, which enhances its inhibitory effects on voluntary uterine contractions and antagonistic effects on acetylcholine-induced spasmodic uterine contractions, thus alleviating spasmodic contractions of the uterus (He et al., 2021). Honey-roasted GU improves blood circulation, boosts immunity, and enhances the palatability of medications (Xu YL. et al., 2020). Stir-baking increases the content of polysaccharides in AM, which form the material basis for the spleen-fortifying and diarrhea-relieving effects of AM and enhance nutrient absorption by the digestive system (Haoyu, 2019).

Among the medicinal materials in the prescriptions for dysmenorrhea, ZJH (Hei Zao, abbreviated as ZJH) and ZJ (Da Zao, abbreviated as ZJ) showed the highest daily doses. There were also considerable differences in the dose level of ZJH and ZJ across different TCM pharmacies, with the adopted dose ranging from 8 to 94 g for ZJH and 6–45 for ZJ. This may be attributed to the fact that both ZJH and ZJ are sweet-tasting, fruit-derived medicinal materials with a lack of toxicity and strict dose limits. Therefore, the dose levels of these materials are largely determined by the preferences of the various TCM pharmacies. Our results also showed that the dose level of HP (15.12 g/day) in the prescriptions for dysmenorrhea was higher than that of the other medicinal materials, but still within the reasonable dose range of 9–30 g as stated in TCM-related pharmacopeias.

## 5 Limitations and Future Works

The study was a field investigation that only collected prescriptions currently sold by TCM pharmacies. The first limitation was that the therapeutic efficacy for these prescriptions was not surveyed. The study only identified the core medicinal materials used for dysmenorrhea, with their doses and processing methods of common usage. The study did not evaluate the activity and efficacy for these medicinal materials. The second limitation was that the network diagram only shows the prescription relationship between the medicinal materials, but not the pharmacological interaction between them. In future works, we will clarify the therapeutic effects of prescriptions for dysmenorrhea by both interviewing the customers and performing clinical trials. The core medicinal materials can be combined to form a new formula based on the data generated by the study. This new formula could become a new product, but its efficacy and safety need to be tested.

## 6 Conclusion

In the present study, an ethnopharmacological survey of prescriptions for dysmenorrhea from TCM pharmacies across Taiwan was performed for the first time. Our results will be beneficial towards the preservation of important knowledge regarding prescriptions for dysmenorrhea in Taiwan. Although the modern pharmacological effects and the processing methods of the component materials have been collated and documented in this study, further in-depth research remains necessary. The results of this study may also serve as reference for the development of naturally derived medications for the treatment of dysmenorrhea. Given that TCM pharmacies may completely disappear in the near future, it is imperative to hasten our efforts in documenting traditional medical knowledge and adopting the necessary measures to preserve the techniques and knowledge passed down in these pharmacies.

## 7 Contributions of This Study

TCM pharmacies are among the most iconic traditional medical settings of Taiwan. Treatment experiences passed down from one generation to another in these pharmacies have contributed to a wealth of prescriptions for dysmenorrhea, which have achieved significant therapeutic effects in countless Taiwanese women. The present study is the first to report an ethnopharmacological survey of prescriptions for dysmenorrhea of TCM pharmacies in Taiwan, and our results can contribute to the documentation, analysis, and retention of medical knowledge related to dysmenorrhea. In addition to collating data on the frequently used medicinal material combinations, TCM property and flavor, and traditional effects of the materials, we also performed a literature search of relevant pharmacological studies to determine the modern pharmacological effects of these materials related to the relief of dysmenorrhea. The doses of the frequently used medicinal materials have also been recorded in this study, which may serve as a reference for the clinical use of these materials by TCM practitioners. Moreover, the frequently used core medicinal material pairs in prescriptions for dysmenorrhea were determined, and a network analysis was performed to provide a network of core medicinal materials used in TCM pharmacy prescriptions for dysmenorrhea. Therefore, the present study can contribute to the documentation and passing down of traditional medical knowledge related to TCM pharmacy prescriptions for dysmenorrhea.

## Data Availability

The original contributions presented in the study are included in the article/Supplementary Material, further inquiries can be directed to the corresponding authors.
