# Supplementary material for: Ethnopharmacological Survey of Traditional Chinese Medicine Pharmacy Prescriptions for Dysmenorrhea
Source: Front Pharmacol. 2021 Dec 21;12:746777. doi: 10.3389/fphar.2021.746777 (PMC8724257; doi:10.3389/fphar.2021.746777)
Supplement: Supplementary file 1 [file DataSheet1.docx]

Supplementary Data

**Table S1.** Basic data of the 77 medicinal materials used in prescriptions for dysmenorrhea

|  | Scientific name | Abbreviation | The Plant List | Family | Local name/Chinese name | Source | Parts used | Usage | Property and flavor | Dosage | RFC |
| --- | --- | --- | --- | --- | --- | --- | --- | --- | --- | --- | --- |
| 1 | *Angelica sinensis* (Oliv.) Diels | AS | *Angelica sinensis* (Oliv.) Diels | Umbelliferae | Dang Guei / Dang Gui(當歸) | Angelicae sinensis radix | dried root | Tonifying and replenishing medicinal (Blood tonifying medicinal). | Warm; sweet and pungent. | 5-15 g | 0.96 |
| 2 | *Achyranthes bidentata* Blume | AB | *Achyranthes bidentata* Blume | Amaranthaceae | Niou Si / Niu Xi(懷牛膝) | Achyranthis bidentatae radix | dried root | Blood-regulating medicinal (Blood-activating and stasis-dispelling medicinal). | Neutral; bitter and sour. | 5-15 g. | 0.04 |
| 3 | *Alisma plantago-aquatica* L. subsp. *orientale* (Sam.) Sam. | AP | *Alisma plantago-aquatica* L. | Alismataceae | Ze Sie / Ze Xie(澤瀉) | Alismatis rhizoma | dried rhizome | Dampness-dispelling medicinal (Dampnessdraining diuretic medicinal). | Cold; sweet and bland. | 6-12 g. | 0.02 |
| 4 | Amomum villosum Lour. | AV | Amomum villosum Lour. | Zingiberaceae | Sha Ren / Sha Ren(砂仁) | Amomi fructus | dried ripe fruit | Dampness-dispelling medicinal (Dampnessresolving with aroma medicinal). | Warm; pungent. | 3-7.5 g. | 0.04 |
| 5 | *Artemisia argyi* H.Lév. et Vaniot | AA | *Artemisia argyi* H.Lév. & Vaniot | Compositae | Ai Ye / Ai Ye(艾草) | Artemisiae argyi folium | dried leaf | Blood-regulating medicinal (Hemostatic medicinal). | Warm; pungent and bitter. | 3-10 g | 0.01 |
| 6 | *Artemisia lactiflora* Wall. ex DC. | AL | *Artemisia lactiflora* Wall. ex DC. | Compositae | Liou Ji Nu/Liu Ji Nu(劉寄奴) | Artemisiae lactiflorae herba | dried aerial part | Blood-regulating medicinal (Blood-activating and stasis-dispelling medicinal). | Warm; bitter. | 6-9 g. | 0.01 |
| 7 | *Asparagus cochinchinensis* (Lour.) Merr. | AC | *Asparagus cochinchinensis* (Lour.) Merr. | Liliaceae | Tian Men Dong / Tian Men Dong(天門冬) | Asparagi radix | dried root | Tonifying and replenishing medicinal (Yin tonifying medicinal). | Cold; sweet and bitter. | 6-12 g | 0.01 |
| 8 | *Astragalus membranaceus* (Fisch.) Bunge var. *mongholicus* (Bunge) P.K.Hsiao | AM | *Astragalus membranaceus* (Fisch.) Bunge | Leguminosae | Huang Ci / Huang Qi(黃耆) | Astragali radix | dried root | Tonifying and replenishing medicinal (Qi tonifying medicinal). | Mild warm; sweet. | 9-30 g. | 0.01 |
| 9 | *Atractylodes chinensis* (DC.) Koidz. | AC | *Atractylodes chinensis* (Bunge) Koidz. | Compositae | Cang Jhu / Cang Zhu(蒼朮) | Atractylodis rhizoma | dried rhizome | Dampness-dispelling medicinal (Dampnessresolving with aroma medicinal). | Warm; pungent and bitter. | 3-9 g. | 0.01 |
| 10 | *Atractylodes macrocephala*  Koidz. | AM | *Atractylodes macrocephala* Koidz. | Compositae | Bai Jhu / Bai Zhu(白朮) | Atractylodis macrocephalae rhizoma | dried rhizome | Tonifying and replenishing medicinal (Qi tonifying medicinal). | Warm; bitter and sweet. | 6-15 g | 0.25 |
| 11 | *Aucklandia lappa* Decne. | AL | *Aucklandia lappa* DC. | Compositae | Mu Siang / Mu Xiang(木香) | Aucklandiae radix | dried root | Qi-regulating medicinal. | Warm; pungent and bitter. | 1.5-6 g. | 0.01 |
| 12 | *Bupleurum chinense* DC | BC | *Bupleurum chinense* DC. | Umbelliferae | Chai Hu / Chai Hu(柴胡) | Bupleuri radix | dried root | Exterior-releasing medicinal (Pungent-cold exterior-releasing medicinal). | Mild cold; pungent and bitter. | 3-10 g. | 0.02 |
| 13 | *Carthamus tinctorius* L. | CaT | *Carthamus tinctorius* L. | Compositae | Hong Hua / Hong Hua(紅花) | Carthami flos | dried tubular flower | Blood-regulating medicinal (Blood-activating and stasis-dispelling medicinal). | Warm; pungent. | 3-10 g | 0.10 |
| 14 | *Cervus nippon* Temminck | CT | *Cervus nippon* Temminck or *Cervus elaphus* Linnaeus | Cervidae | Lurong /(鹿茸) | Cervus nippon Temminck | young non-ossified velvet antler | Tonifying and replenishing medicinal (Yang tonifying medicinal) | Warm, sweet., salty， |  | 0.02 |
| 15 | *Chaenomeles speciosa* (Sweet) Nakai | CS | *Chaenomeles speciosa* (Sweet) Nakai | Rosaceae | Mu Gua / Mu Gua(木瓜) | Chaenomelis fructus | dried ripe fruit | Dampness-dispelling medicinal (Wind-dampnessdispelling medicinal). | Warm; sour. | 6-12 g | 0.02 |
| 16 | *Cibotium barometz* (L.) J.Sm. | CB | *Cibotium barometz* (L.) J.Sm. | Dicksoniaceae | Gou Ji / Gou Ji(狗脊) | Cibotii rhizoma | dried rhizome | Tonifying and replenishing medicinal (Yang tonifying medicinal). | Warm; bitter and sweet. | 6-12 g. | 0.03 |
| 17 | *Cinnamomum cassia* (L.) J.Presl | CCT | *Cinnamomum cassia* (Nees & T.Nees) J.Presl | Lauraceae | Guei Jhih / Gui Zhi(桂枝) | Cinnamomi ramulus | dried twig | Exterior-releasing medicinal (Pungent-warm exterior-releasing medicinal). | Warm; pungent and sweet. | 3-10 g | 0.59 |
| 18 | *Cinnamomum cassia* (L.) J.Presl | CCB | *Cinnamomum cassia* (Nees & T.Nees) J.Presl | Lauraceae | Rou Guei / Rou Gui(肉桂) | Cinnamomi cortex | dried bark of trunk | Interior-warming medicinal. | Highly hot; pungent and sweet. | 1-5 g | 0.28 |
| 19 | *Cinnamomum japonicum* Sieb. | CJ | *Cinnamomum japonicum* Siebold | Lauraceae | Guei Zi / Gui Zi(桂子) | Cinnamomum japonicum Sieb.[C.pedunculatum Nees;C.chekiangense | dried seed | Interior-warming medicinal. | Pungent; sweet and warm | 3-6g | 0.05 |
| 20 | *Citrus reticulata* Blanco | CR | *Citrus reticulata* Blanco | Rutaceae | Chen Pi / Chen Pi(陳皮) | Citri reticulatae pericarpium | dried pericarp of the ripe fruit | Qi-regulating medicinal. | Warm; bitter and pungent. | 3-11.5 g. | 0.02 |
| 21 | *Codonopsis pilosula (Franch.) Nannf.* | CP | Codonopsis pilosula (Franch.) Nannf. | Campanulaceae | Dang Shen / Dang Shen(黨參) | Codonopsis radix | dried root | Tonifying and replenishing medicinal (Qi tonifying medicinal). | Neutral; sweet. | 9-30 g | 0.40 |
| 22 | *Cordyceps militaris* | CM | *Clavaria militaris* L. | Clavicipitaceae | Bei Chong Cao (北蟲草) | Cordyceps militaris |  | Tonifying and replenishing medicinal (Yangtonifying  medicinal). | Warm; sweet. | 3-10 g. | 0.01 |
| 23 | *Cornus officinalis* Siebold et Zucc. | CO | *Cornus officinalis* Siebold & Zucc. | Cornaceae | Shan Jhu Yu / Shan Zhu Yu山茱萸 (棗肉) | Corni sarcocarpium | dried ripe sarcocarp | Tonifying and replenishing medicinal (Yin tonifying medicinal). | Mild warm; sour and astringent. | 5-12 g. | 0.01 |
| 24 | *Corydalis yanhusuo*W.T.Wang | CY | *Corydalis yanhusuo (*Y.H.Chou & Chun C.Hsu) W.T.Wang ex Z.Y.Su & C.Y.Wu | Papaveraceae | Yan Hu Suo / Yan Hu Su (延胡索) | Corydalis rhizoma | dried tuber | Blood-regulating medicinal (Blood-activating and stasis-dispelling medicinal). | Warm; pungent and bitter. | 3-12 g | 0.10 |
| 25 | *Curcuma phaeocaulis* Valeton | CP | *Curcuma phaeocaulis* Valeton | Zingiberaceae | E Jhu / E Zhu (莪朮) | Curcumae rhizoma | dried rhizome | Blood-regulating medicinal (Blood-activating and stasis-dispelling medicinal). | Warm; pungent and bitter. | 6-9 g. | 0.02 |
| 26 | *Cyathula officinalis* Kuan | CO | *Cyathula officinalis* K.C.Kuan | Amaranthaceae | Chuan Niou Si / Chuan Niu Xi川牛膝 (台灣稱杜牛膝) | Cyathulae radix | dried root | Blood-regulating medicinal (Blood-activating and stasis-dispelling medicinal). | Neutral; bitter and sour. | 3-10 g. | 0.02 |
| 27 | *Cyperus rotundus* L. | CR | *Cyperus rotundus* L*.* | Cyperaceae | Siang Fu / Xiang Fu (香附) | Cyperi rhizoma | dried rhizome | Qi-regulating medicinal. | Neutral; pungent, mild bitter and mild sweet. | 6-11.5 g | 0.15 |
| 28 | *Dimocarpus longgana* Lour. | DL | *Dimocarpus longan* Lour. | Sapindaceae | LONG YAN (龍眼乾) | Euphoria longan (Lour.) Steud. | dried ripe pulp | Tonifying and replenishing medicinal (Qi tonifying medicinal). | Neutral,sweet and bland. | 6-9 g | 0.01 |
| 29 | *Dioscorea opposita* Thunb. | DO | *Dioscorea opposita* Thunb. | Dioscoreaceae | Shan Yao / Shan Yao(山藥) | Dioscoreae rhizoma | dried rhizome | Tonifying and replenishing medicinal (Qi tonifying medicinal). | Neutral; sweet. | 10-30 g | 0.05 |
| 30 | *Dipsacus inermis* Wall. | DI | *Dipsacus inermis* Wall. | Dipsacaceae | Syu Duan / Xu Duan續斷(六汗) | Dipsaci radix | dried root | Tonifying and replenishing medicinal (Yang tonifying medicinal). | Mild warm; bitter and pungent. | 9-15 g | 0.07 |
| 31 | *Drynaria roosii* Nakaike | DR | *Drynaria fortune*i (Kunze ex Mett.) J.Sm. | Polypodiaceae | Gu Suei Bu/Gu Sui Bu (骨碎補) | Drynariae rhizoma | dried rhizome | Tonifying and replenishing medicinal (Yang tonifying medicinal). | Warm; bitter. | 3-12 g | 0.05 |
| 32 | *Equus asinus* L. | EA | *Equus asinus* L | Equidae | (阿膠) | Equus asinus L. | The solid gelati made from the skin of *Equus asinus* L, via a process of boiling and concentrating. | Tonifying and replenishing blood tonifying medicinal (Hemostatic medicinal) | Neutral,sweet |  | 0.02 |
| 33 | *Eriocaulon buergerianum* Körn | EB | *Eriocaulon buergerianum* Körn. | Eriocaulaceae | Gu Jing Cao / Gu Jing Cao(穀精子) | Eriocauli flos | dried inflorescence | Heat-clearing medicinal (Heat-clearing and firepurging medicinal). | Neutral; pungent and sweet. | 4.5-15 g. | 0.02 |
| 34 | *Eucommia ulmoides* Oliv. | EU | *Eucommia ulmoides* Oliv. | Eucommiaceae | Du Jhong / Du Zhong(杜仲) | Eucommiae cortex | dried bark of trunk | Tonifying and replenishing medicinal (Yang tonifying medicinal). | Warm; sweet. | 6-15 g | 0.38 |
| 35 | Euodia ruticarpa (A.Juss.) Benth. | ER | *Euodia ruticarpa* (A. Juss.) Benth. | Rutaceae | Wu Jhu Yu / Wu Zhu Yu(吳茱萸) | Euodiae fructus | dried and almost ripe fruit | Interior-warming medicinal. | Hot; pungent and bitter. | 1.0-7.5 g | 0.01 |
| 36 | *Foeniculum vulgare* Mill. | FV | *Foeniculum vulgare* Mill. | Umbelliferae | Siao Huei Siang / Xiao Hui Xiang(小茴香) | Foeniculi fructus | dried ripe fruit | Interior-warming medicinal. | Warm; pungent. | 3-11.5 g. | 0.01 |
| 37 | *Ginkgo biloba* L. | GB | *Ginkgo biloba* L. | Ginkgoaceae | Bai Guo / Bai Guo(白果) | Ginkgo semen | dried ripe seed | Phlegm-dispelling medicinal (Cough-suppressing and panting-calming medicinal). | Neutral; sweet, bitter and astringent. | 4.5-11.5 g | 0.07 |
| 38 | *Glycyrrhiza uralensis* Fisch. | GU | *Glycyrrhiza uralensis* Fisch. | Leguminosae | Gan Cao / Gan Cao(甘草) | Glycyrrhizae radix et rhizoma | dried root and rhizome | Tonifying and replenishing medicinal (Qi tonifying medicinal). | Neutral; sweet. | 2-11.5 g | 0.45 |
| 39 | *Hedysarum polybotry*s Hand.-Mazz. | HP | *Hedysarum polybotrys* Hand.-Mazz. | Leguminosae | Hong Ci / Hong Qi(紅耆) | Hedysari radix | dried root | Tonifying and replenishing medicinal (Qi tonifying medicinal). | Mild warm; sweet. | 9-30 g | 0.68 |
| 40 | *Imperata cylindrica* (L.) P.Beauv. var. *major* (Nees) C.E.Hubb. | IC | *Imperata cylindrica* (L.) Raeusch. | Gramineae | Bai Mao Gen / Bai Mao Gen(白茅根) | Imperatae rhizoma | dried rhizome | Blood-regulating medicinal (Hemostatic medicinal). | Cold; sweet. | 9-30 g. | 0.01 |
| 41 | *Leonurus japonicus* Houtt. | LJ | *Leonurus japonicus* Houtt. | Labiatae | Yi Mu Cao / Yi Mu Cao(益母草) | Leonuri herba | dried aerial part | Blood-regulating medicinal (Blood-activating and stasis-dispelling medicinal). | Mild cold; bitter and pungent. | 9-30 g. | 0.14 |
| 42 | *Ligusticum chuanxiong* Hort | LiC | *Ligusticum striatum* DC. *.* | Umbelliferae | Chuan Cyong / Chuan Qiong(川芎) | Chuanxiong rhizoma | dried rhizome | Blood-regulating medicinal (Blood-activating and stasis-dispelling medicinal). | Warm; pungent. | 3-10 g | 0.95 |
| 43 | *Lindera aggregata* (Sims) Kosterm. | LA | *Lindera aggregata* (Sims) Kosterm. | Lauraceae | Wu Yao / Wu Yao(烏藥) | Linderae radix | dried root tuber | Qi-regulating medicinal. | Warm; pungent. | 3-11.5 g. | 0.04 |
| 44 | *Lycium chinense* Mill.. | LyC | *Lycium chinense* Mill. | Solanaceae | Gou Ci Zih / Gou Qi Zi(枸杞子) | Lycii fructus | dried ripe fruit | Tonifying and replenishing medicinal (Yin tonifying medicinal). | Neutral; sweet. | 6-15 g | 0.64 |
| 45 | *Lygodium japonicum* (Thunb.) Sw. | LJ | *Lygodium japonicum* (Thunb.) Sw. | Lygodiaceae | Hai Jin Sha / Hai Jin Sha(海金沙) | Lygodii spora | dried ripe spore | Dampness-dispelling medicinal (Dampnessdraining diuretic medicinal). | Cold; sweet and salty. | 6-15 g. | 0.01 |
| 46 | *Melia toosendan* Siebold et Zucc. | MT | *Melia toosendan* Siebold & Zucc. | Meliaceae | Chuan Lian Zih / Chuan Lian Zi(川楝子) | Toosendan fructus | dried ripe fruit | Dampness-dispelling medicinal (Wind-dampnessdispelling medicinal). | Mild cold; bitter. | 6-12 g. | 0.01 |
| 47 | *Nelumbo nucifera* Gaertn. | NN | *Nelumbo nucifera* Gaertn. | Nymphaeaceae | Lian Zih / Lian Zi(蓮子) | Nelumbinis semen | dried ripe seed | Astringent medicinal. | Neutral; sweet and astringent. | 6-15 g. | 0.05 |
| 48 | *Ophiopogon japonicus* (L.f.) Ker Gawl. | OJ | *Ophiopogon japonicus* (Thunb.) Ker Gawl. | Asparagaceae | Mai Men Dong / Mai Men Dong(麥門冬) | Ophiopogonis radix | dried root tuber | Tonifying and replenishing medicinal (Yintonifying medicinal). | Mild cold; sweet and mild bitter. | 6-15 g. | 0.02 |
| 49 | *Oroxylum indicum*(L.) Benth. ex Kurz | OI | *Oroxylum indicum* (L.) Kurz | Bignoniaceae | Mu Hu Dieh / Mu Hu Dieh(木蝴蝶) | Oroxyli semen | dried seed | Heat-clearing medicinal (Heat-clearing and detoxicating medicinal). | Cool; bitter and sweet. | 1-4 g | 0.31 |
| 50 | *Paeonia lactiflora* Pall. | PLR | *Paeonia lactiflora* Pall. | Ranunculaceae | Chih Shao / Chi Shao(赤芍) | Paeoniae rubra radix | dried root | Heat-clearing medicinal (Heat-clearing and blood cooling medicinal). | Mild cold; bitter. | 3-12 g. | 0.03 |
| 51 | *Paeonia lactiflora*Pall. | PL | *Paeonia lactiflora* Pall. | Ranunculaceae | Bai Shao / Bai Shao(白芍) | Paeoniae alba radix | peeled and dried root | Tonifying and replenishing medicinal (Blood tonifying medicinal). | Mild cold; bitter and sour. | 6-15 g | 0.71 |
| 52 | *Paeonia suffruticosa* Andrews | PS | *Paeonia* × suffruticosa Andrews | Ranunculaceae | Mu Dan Pi / Mu Dan Pi(牡丹皮) | Moutan radicis cortex | dried bark of root | Heat-clearing medicinal (Heat-clearing and blood cooling medicinal). | Mild cold; bitter and pungent. | 6-12 g. | 0.03 |
| 53 | *Panax ginseng* C.A.Mey. | PG | *Panax ginseng* C.A.Mey. | Araliaceae | Ren Shen / Ren Shen(人參) | Ginseng radix et rhizoma | dried root and rhizome | Tonifying and replenishing medicinal (Qi tonifying medicinal). | Mild warm; sweet and mild bitter. | 3-11.5 g. | 0.03 |
| 54 | *Panax ginseng* C.A.Mey. | PG | *Panax ginseng* C.A.Mey. | Araliaceae | Ren Shen / Ren Shen(吉林參) | Ginseng radix et rhizoma | dried root and rhizome | Tonifying and replenishing medicinal (Qi tonifying medicinal). | Mild warm; sweet and mild bitter. | 3-11.5 g. | 0.01 |
| 55 | *Panax notoginseng* (Burkill) F.H.Chen | PN | *Panax notoginseng* (Burkill) F.H.Chen | Araliaceae | San Ci / San Qi(三七) | Notoginseng radix et rhizoma | dried root and rhizome | Blood-regulating medicinal (Hemostatic medicinal). | Mild warm; sweet and mild bitter. | 3-11.5 g | 0.01 |
| 56 | *Panax quinquefolius* L. | PQ | *Panax quinquefolius* L. | Araliaceae | Si Yang Shen / Xi Yang Shen(西洋參) | Panacis quinquefolii radix | dried root | Tonifying and replenishing medicinal (Qi tonifying medicinal). | Cool; sweet and mild bitter. | 3-12 g. | 0.02 |
| 57 | *Phyla nodiflora* （L.）Greene [*Lippia nodiflora* （L.）Rich.] | PN | *Phyla nodiflora (L.)* Greene | Verbenaceae | 鴨舌癀(石莧) | Knottedflower Phyla |  |  |  |  | 0.02 |
| 58 | *Pinellia ternata* (Thunb.) Breitenb. | PT | *Pinellia ternata* (Thunb.) Makino | Araceae | Ban Sia / Ban Xia(半夏) | Pinelliae rhizoma | dried tuber | Phlegm-dispelling medicinal (Dampness and phlegm eliminating medicinal). | Warm; pungent; toxic. | 3-11.5 g, | 0.01 |
| 59 | *Polygonatum odoratum* (Mill.) Druce | PO | *Polygonatum odoratum* (Mill.) Druce | Asparagaceae | Yu Jhu / Yu Zhu(玉竹) | Polygonati odorati rhizoma | dried rhizome | Tonifying and replenishing medicinal (Yin tonifying medicinal). | Mild cold; sweet. | 6-12 g | 0.04 |
| 60 | *Polygonum multiflorum* Thunb. | PM | *Reynoutria multiflora* (Thunb.) Moldenke . | Polygonaceae | He Shou Wu / He Shou Wu(何首烏) | Polygoni multiflori radix | dried root tuber | Tonifying and replenishing medicinal (Blood tonifying medicinal). | Mild warm; bitter, sweet and astringent. | 6-15 g. | 0.03 |
| 61 | Poria cocos (Schwein.) F.A.Wolf | PC | *Poria cocos (Schwein.) F.A.Wolf* | Polyporaceae | Fu Ling / Fu Ling(茯苓) | Poria | dried sclerotium | Dampness-dispelling medicinal (Dampness draining diuretic medicinal). | Neutral; sweet and bland. | 9-30 g | 0.27 |
| 62 | *Portulaca oleracea* L. | Pol | *Portulaca oleracea* L. | Portulacaceae | Ma Chih Sian / Ma Chi Xian(馬齒莧) | Portulacae herba | dried aerial part | Heat-clearing medicinal (Heat-clearing and detoxicating medicinal). | Cold; sour. | 9-15 g. | 0.01 |
| 63 | Prunus persica (L.) Batsch | PP | *Prunus persica* (L.) Batsch | Rosaceae | Tao Ren / Tao Ren(桃仁) | Persicae semen | dried ripe seed | Blood-regulating medicinal (Blood-activating and stasis-dispelling medicinal). | Neutral; bitter and sweet. | 4.5-10 g | 0.21 |
| 64 | *Pteroxygonum giraldii* Dammer & Diels | PG | *Pteroxygonum giraldii* Dammer & Diels | Polygonaceae | Yi Liao / Yi Liao(翼蓼) | Pteroxygonum giraldii Damm. et Diels | dried root tuber | Clear heat, detoxify, stop bleeding | Sour,bitter,astringent.cool | 7.5-18.75 g. | 0.02 |
| 65 | *Rehmannia glutinosa*  Libosch. | RG | *Rehmannia glutinosa* (Gaertn.) DC | Scrophulariaceae | Di Huang / Di Huang(地黃) | Rehmanniae radix | fresh or dried root | Heat-clearing medicinal (Heat-clearing and blood cooling medicinal). | Cold sweet and bitter. | 9-30 g | 0.79 |
| 66 | *Salvia miltiorrhiza* Bunge | SM | *Salvia miltiorrhiza* Bunge | Labiatae | Dan Shen / Dan Shen(丹參) | Salviae miltiorrhizae radix et rhizoma | dried root and rhizome | Blood-regulating medicinal (Blood-activating and stasis-dispelling medicinal). | Mild cold; bitter. | 5-15 g. | 0.02 |
| 67 | *Sparganium stoloniferum* (Graebn.) Buch.-Ham. ex Juz. | SS | *Sparganium stoloniferum* (Buch.-Ham. ex Graebn.) Buch.-Ham. ex Juz. | Sparganiaceae | San Ling / San Ling(三稜) | Sparganii rhizoma | dried tuber | Blood-regulating medicinal (Blood-activating and stasis-dispelling medicinal). | Neutral; pungent and bitter. | 4.5-11.5 g. | 0.01 |
| 68 | *Spatholobus suberectus* Dunn | Ssu | *Spatholobus suberectus* Dunn | Leguminosae | Ji Sie Teng / Ji Xie Teng(雞血藤) | Spatholobi caulis | dried lianoid stem | Blood-regulating medicinal (Blood-activating and stasis-dispelling medicinal). | Warm; bitter and sweet. | 9-15 g. | 0.02 |
| 69 | *Stachys sieboldii* Miq. | Ssi | *Stachys sieboldii* Miq. | Labiatae | (草石蠶) | Chinese Artichoke，Artichoke Betony Chorogi，Japanese Artichoke | dried rhizome |  |  |  | 0.01 |
| 70 | *Strobilanthes forrestii* Diels, | SF | *Strobilanthes forrestii* Diels | Acanthaceae | Wei Niou Si / Wei Niu Xi味牛膝(台灣稱川牛膝) | Strobilanthes forrestii Diels | dried root and rhizome | Blood-regulating medicinal (Blood-activating and stasis-dispelling medicinal). | Neutral; bitter and sour. |  | 0.02 |
| 71 | *Taxillus chinensis* (DC.) Danser | TC | *Taxillus chinensis* (DC.) Danser | Loranthaceae | Sang Ji Sheng / Sang Ji Sheng(桑寄生) | taxilli herba | dried stem and branch with leaf | Dampness-dispelling medicinal (Wind-dampnessdispelling medicinal). | Neutral; sweet and bitter. | 9-15 g. | 0.01 |
| 72 | *Tournefortia sarmentosa* Lamarck | TS | *Tournefortia sarmentosa* Lam. | Boraginaceae | (倒爬麒麟) | Tournefortia sarmentosa | dried rhizome | Blood-activating and detoxicating medicinal | Neutral; bitter,astringent |  | 0.01 |
| 73 | *Trichosanthes kirilowii* Maxim. | TK | *Trichosanthes kirilowii* Maxim. | Cucurbitaceae | Gua Lou Gen / Gua Lou Gen天花(栝樓根) | Trichosanthis radix | dried root | Heat-clearing medicinal (Heat-clearing and firepurging medicinal). | Mild cold; sweet and mild bitter. | 10-15 g. | 0.01 |
| 74 | *Zingiber officinale*Roscoe | ZO | *Zingiber officinale* Roscoe | Zingiberaceae | Gan Jiang / Gan Jiang(乾薑) | Zingiberis rhizoma | dried rhizome | Interior-warming medicinal. | Hot; pungent. | 3-9 g | 0.17 |
| 75 | *Ziziphus jujuba* Mill. | ZJ | *Ziziphus jujuba* Mill. | Rhamnaceae | Da Zao / Da Zao(大棗) | Jujubae fructus | dried ripe fruit | Tonifying and replenishing medicinal (Qi tonifying medicinal). | Warm; sweet. | 6-30 g | 0.36 |
| 76 | Ziziphus jujuba Mill. | ZJH | Ziziphus jujuba Mill. | Rhamnaceae | Hei Zao / Hei Zao(黑棗) | Jujubae fructus | dried ripe fruit | Tonifying and replenishing medicinal (Qi tonifying medicinal). | Warm; sweet. | 6-30 g | 0.30 |
| 77 | *Ziziphus mauritiana* Lam. | ZM | *Ziziphus mauritiana* Lam*.* | Rhamnaceae | (蜜棗) | Jujubae fructus | dried ripe fruit |  | Neutral,sweet |  | 0.01 |

**Table S2.** Doses of medicinal materials frequently used in prescriptions for dysmenorrhea

| Medicine(g) | RFC | Mean | SD | Min | First quartile  (Q1) | Median | Third quartile  (Q3) | Max | IQR=Q3-Q1 | Upper limit Q3+1.5IQR | Lower limit Q1-1.5IQR | Relative ratio* |
| --- | --- | --- | --- | --- | --- | --- | --- | --- | --- | --- | --- | --- |
| AS | 0.96 | 10.84 | 3.80 | 3.00 | 8.80 | 11.00 | 13.50 | 60.00 | 4.70 | 20.55 | 1.75 | 1.00 |
| LiC | 0.95 | 10.94 | 4.17 | 1.80 | 7.50 | 10.85 | 13.95 | 38.30 | 6.45 | 23.63 | -2.18 | 1.01 |
| RG | 0.79 | 12.22 | 3.05 | 4.50 | 11.00 | 12.30 | 15.00 | 61.60 | 4.00 | 21.00 | 5.00 | 1.13 |
| PL | 0.71 | 11.04 | 4.52 | 1.70 | 7.50 | 11.30 | 13.80 | 27.60 | 6.31 | 23.26 | -1.96 | 1.02 |
| HP | 0.68 | 15.12 | 7.24 | 4.80 | 10.55 | 14.40 | 20.15 | 44.00 | 9.60 | 34.55 | -3.85 | 1.40 |
| LyC | 0.64 | 14.17 | 4.91 | 5.40 | 10.30 | 13.80 | 17.90 | 58.50 | 7.60 | 29.30 | -1.10 | 1.31 |
| CCT | 0.59 | 10.98 | 5.37 | 1.20 | 7.15 | 10.40 | 14.95 | 54.50 | 7.80 | 26.65 | -4.55 | 1.01 |
| GU | 0.45 | 6.20 | 3.14 | 0.90 | 4.10 | 6.00 | 8.70 | 34.90 | 4.60 | 15.60 | -2.80 | 0.57 |
| CP | 0.40 | 9.96 | 3.80 | 2.60 | 7.72 | 10.05 | 12.78 | 26.50 | 5.06 | 20.37 | 0.13 | 0.92 |
| EU | 0.38 | 13.17 | 5.14 | 3.80 | 10.20 | 13.30 | 18.13 | 48.30 | 7.93 | 30.01 | -1.69 | 1.21 |
| ZJ | 0.36 | 16.26 | 5.21 | 6.60 | 13.75 | 16.30 | 20.80 | 45.00 | 7.05 | 31.38 | 3.18 | 1.50 |
| OI | 0.31 | 3.09 | 2.04 | 0.30 | 1.80 | 2.60 | 4.30 | 11.20 | 2.50 | 8.05 | -1.95 | 0.29 |
| ZJH | 0.30 | 25.18 | 8.48 | 8.40 | 20.50 | 28.65 | 33.15 | 94.40 | 12.65 | 52.13 | 1.52 | 2.32 |
| CCB | 0.28 | 5.53 | 2.69 | 0.70 | 3.30 | 6.20 | 7.70 | 16.60 | 4.40 | 14.30 | -3.30 | 0.51 |
| PC | 0.27 | 10.51 | 3.26 | 3.70 | 8.33 | 10.00 | 11.95 | 17.10 | 3.63 | 17.39 | 2.89 | 0.97 |
| AM | 0.25 | 7.99 | 2.29 | 2.70 | 6.65 | 9.00 | 10.15 | 32.40 | 3.50 | 15.40 | 1.40 | 0.74 |
| PP | 0.21 | 4.81 | 2.43 | 1.00 | 3.90 | 5.60 | 7.00 | 12.20 | 3.10 | 11.65 | -0.75 | 0.44 |
| ZO | 0.17 | 3.48 | 1.74 | 0.60 | 2.70 | 4.14 | 5.00 | 10.00 | 2.30 | 8.45 | -0.75 | 0.32 |
| CR | 0.15 | 9.36 | 3.30 | 4.00 | 6.95 | 9.10 | 11.75 | 15.40 | 4.80 | 18.95 | -0.25 | 0.86 |
| LJ | 0.14 | 11.06 | 4.46 | 5.00 | 7.63 | 11.20 | 12.83 | 18.80 | 5.20 | 20.63 | -0.17 | 1.02 |
| CaT | 0.10 | 4.87 | 1.98 | 2.40 | 3.43 | 5.05 | 6.95 | 14.60 | 3.53 | 12.24 | -1.86 | 0.45 |
| CY | 0.10 | 9.19 | 3.59 | 5.40 | 7.05 | 7.75 | 10.83 | 16.20 | 3.78 | 16.49 | 1.39 | 0.85 |

Relative ratio: Relative dose of a medicinal material when the dose for AS is set as 1

AS, *Angelica sinensis* (Oliv.) Diels; AM, *Atractylodes macrocephala* Koidz.;CCT, *Cinnamomum cassia* (L.) J.Presl ; CPM, *Codonopsis pilosula* (Franch.) Nannf. var. *modesta* (Nannf.) L.T.Shen ;CCB, *Cinnamomum cassia* (L.) J.Presl; CR, *Cyperus rotundus* L.; CaT, *Carthamus tinctorius* L.; CY, *Corydalis yanhusuo*W.T.Wang.; EU, *Eucommia ulmoides* Oliv.; GI, *Glycyrrhiza inflata*Batalin ; HP, *Hedysarum polybotrys* Hand.-Mazz.; LiC, *Ligusticum chuanxiong* Hort; LyC, *Lycium chinense*Mill.; LJ, *Leonurus japonicus* Houtt.; OI, *Oroxylum indicum* (L.) Benth. ex Kurz ; PL, *Paeonia lactiflora*Pall.; PC, *Poria cocos*(Schwein.) F.A.Wolf ; PP, *Prunus persica* (L.) Batsch ; RG, *Rehmannia glutinosa* Libosch.; ZJ, *Ziziphus jujuba* Mill.; ZJH, *Ziziphus jujuba* Mill. ; ZO, *Zingiber officinale*Roscoe.

**Table S3.** Medicinal material combinations

|  | Medicinal material combinations | Frequency |
| --- | --- | --- |
| 1 | LiC－CCT | 58 |
| 2 | LiC－ AS | 92 |
| 3 | LiC－ PL | 69 |
| 4 | LiC－ LyC | 63 |
| 5 | LiC－ RG | 73 |
| 6 | LiC－ HP | 65 |
| 7 | CCT－ AS | 57 |
| 8 | LyC－ CCT | 50 |
| 9 | CCT－ RG | 50 |
| 10 | PL－ AS | 70 |
| 11 | LyC－ AS | 62 |
| 12 | AS－ RG | 75 |
| 13 | AS－ HP | 66 |
| 14 | PL－ LyC | 55 |
| 15 | PL－ RG | 67 |
| 16 | PL－ HP | 52 |
| 17 | LyC－ RG | 60 |
| 18 | LyC－ HP | 56 |
| 19 | RG－ HP | 57 |
| 20 | LiC－ CCT－ AS | 57 |
| 21 | LiC－ LyC－ CCT | 50 |
| 22 | LiC－ CCT－ RG | 50 |
| 23 | LiC－ PL－ AS | 69 |
| 24 | LiC－ LyC－ AS | 62 |
| 25 | LiC－ AS－ RG | 73 |
| 26 | LiC－ AS－ HP | 64 |
| 27 | LiC－ PL－ LyC | 55 |
| 28 | LiC－ PL－ RG | 66 |
| 29 | LiC－ PL－ HP | 51 |
| 30 | LiC－ LyC－ RG | 60 |
| 31 | LiC－ LyC－ HP | 56 |
| 32 | LiC－ RG－ HP | 56 |
| 33 | CCT－ AS－ RG | 50 |
| 34 | PL－ LyC－ AS | 55 |
| 35 | PL－ AS－ RG | 67 |
| 36 | PL－ AS－ HP | 52 |
| 37 | LyC－ AS－ RG | 60 |
| 38 | LyC－ AS－ HP | 55 |
| 39 | AS－ RG－ HP | 57 |
| 40 | PL－ LyC－ RG | 55 |
| 41 | PL－ RG－ HP | 52 |
| 42 | LyC－ RG－ HP | 53 |
| 43 | LiC－ CCT－AS－ RG | 50 |
| 44 | LiC－ PL－ LyC－ AS | 55 |
| 45 | LiC－ PL－AS－ RG | 66 |
| 46 | LiC－ PL－ AS－ HP | 51 |
| 47 | LiC－ LyC－ AS－ RG | 60 |
| 48 | LiC－ LyC－ AS－ HP | 55 |
| 49 | LiC－ AS－ RG－ HP | 56 |
| 50 | LiC－ PL－ LyC－ RG | 55 |
| 51 | LiC－ PL－ RG－ HP | 51 |
| 52 | LiC－ LyC－ RG－ HP | 53 |
| 53 | PL－ LyC－AS－ RG | 55 |
| 54 | PL－ AS－ RG－ HP | 52 |
| 55 | LyC－ AS－ RG－ HP | 53 |
| 56 | LiC－ PL－ LyC－ AS－ RG | 55 |
| 57 | LiC－ PL－ AS－ RG－ HP | 51 |
| 58 | LiC－ LyC－ AS－ RG－ HP | 53 |

**Table S4.** Analysis of association rules of the 77 medicinal materials

|  | Associations of medicinal material pairs | Confidence |
| --- | --- | --- |
| 1 | CCT → LiC | 1 |
| 2 | AS → LiC | 0.97 |
| 3 | LiC → AS | 0.98 |
| 4 | PL → LiC | 0.99 |
| 5 | LyC → LiC | 1 |
| 6 | RG → LiC | 0.97 |
| 7 | HP → LiC | 0.97 |
| 8 | CCT → AS | 0.98 |
| 9 | PL → AS | 1 |
| 10 | LyC → AS | 0.98 |
| 11 | RG → AS | 1 |
| 12 | HP → AS | 0.99 |
| 13 | PL → RG | 0.96 |
| 14 | LyC → RG | 0.95 |
| 15 | CCT－ AS → LiC | 1 |
| 16 | LiC－ CCT → AS | 0.98 |
| 17 | CCT →LiC－ AS | 0.98 |
| 18 | PL－ AS → LiC | 0.99 |
| 19 | LiC－ PL → AS | 1 |
| 20 | PL → LiC－ AS | 0.99 |
| 21 | LyC－ AS → LiC | 1 |
| 22 | LiC－ LyC → AS | 0.98 |
| 23 | LyC → LiC－AS | 0.98 |
| 24 | AS－ RG → LiC | 0.97 |
| 25 | LiC－ RG → AS | 1 |
| 26 | RG → LiC－ AS | 0.97 |
| 27 | AS－ HP → LiC | 0.97 |
| 28 | LiC－ HP → AS | 0.98 |
| 29 | HP → LiC－ AS | 0.96 |
| 30 | PL－ LyC → LiC | 1 |
| 31 | PL－ RG → LiC | 0.99 |
| 32 | LiC－ PL → RG | 0.96 |
| 33 | PL－ HP → LiC | 0.98 |
| 34 | LyC－ RG → LiC | 1 |
| 35 | LiC－ LyC → RG | 0.95 |
| 36 | LyC → LiC－ RG | 0.95 |
| 37 | LyC－ HP → LiC | 1 |
| 38 | RG－ HP → LiC | 0.98 |
| 39 | PL－ LyC → AS | 1 |
| 40 | PL－ RG → AS | 1 |
| 41 | PL－ AS → RG | 0.96 |
| 42 | PL → AS－ RG | 0.96 |
| 43 | PL－ HP → AS | 1 |
| 44 | LyC－ RG → AS | 1 |
| 45 | LyC－ AS → RG | 0.97 |
| 46 | LyC → AS－ RG | 0.95 |
| 47 | LyC－ HP → AS | 0.98 |
| 48 | RG－ HP → AS | 1 |
| 49 | PL－ LyC → RG | 1 |
| 50 | PL－ HP → RG | 1 |
| 51 | PL－ LyC－AS → LiC | 1 |
| 52 | LiC－ PL－ LyC → AS | 1 |
| 53 | PL－ LyC → LiC－AS | 1 |
| 54 | PL－ AS－ RG → LiC | 0.99 |
| 55 | LiC－ PL－ RG → AS | 1 |
| 56 | LiC－ PL－ AS → RG | 0.96 |
| 57 | PL－ RG → LiC－AS | 0.99 |
| 58 | PL－ AS－ HP → LiC | 0.98 |
| 59 | LiC－ PL－ HP → AS | 1 |
| 60 | PL－ HP → LiC－AS | 0.98 |
| 61 | LyC－ AS－ RG → LiC | 1 |
| 62 | LiC－ LyC－ RG → AS | 1 |
| 63 | LiC－ LyC－ AS → RG | 0.97 |
| 64 | LyC－ RG → LiC－AS | 1 |
| 65 | LyC－ AS → LiC－ RG | 0.97 |
| 66 | LiC－ LyC → AS－ RG | 0.95 |
| 67 | LyC → LiC－ AS－ RG | 0.95 |
| 68 | LyC－AS－ HP → LiC | 1 |
| 69 | LiC－ LyC－ HP → AS | 0.98 |
| 70 | LyC－ HP → LiC－ AS | 0.98 |
| 71 | AS－ RG－ HP → LiC | 0.98 |
| 72 | LiC－ RG－ HP → AS | 1 |
| 73 | RG－ HP → LiC－ AS | 0.98 |
| 74 | PL－ LyC－ RG → LiC | 1 |
| 75 | LiC－ PL－ LyC → RG | 1 |
| 76 | PL－ LyC → LiC－ RG | 1 |
| 77 | PL－ RG－ HP → LiC | 0.98 |
| 78 | LiC－ PL－ HP → RG | 1 |
| 79 | PL－ HP → LiC－ RG | 0.98 |
| 80 | LyC－ RG－ HP → LiC | 1 |
| 81 | PL－ LyC－ RG → AS | 1 |
| 82 | PL－ LyC－ AS → RG | 1 |
| 83 | PL－ LyC → AS－ RG | 1 |
| 84 | PL－ RG－ HP → AS | 1 |
| 85 | PL－ AS－ HP → RG | 1 |
| 86 | PL－ HP → AS－ RG | 1 |
| 87 | LyC－ RG－ HP → AS | 1 |
| 88 | LyC－ AS－ HP → RG | 0.96 |
| 89 | PL－ LyC－AS－ RG → LiC | 1 |
| 90 | LiC－ PL－ LyC－ RG → AS | 1 |
| 91 | LiC－ PL－ LyC－AS → RG | 1 |
| 92 | PL－ LyC－ RG → LiC－AS | 1 |
| 93 | PL－ LyC－AS → LiC－ RG | 1 |
| 94 | LiC－ PL－ LyC → AS－ RG | 1 |
| 95 | PL－ LyC → LiC－AS－ RG | 1 |
| 96 | PL－ AS－ RG－ HP → LiC | 0.98 |
| 97 | LiC－ PL－ RG－ HP → AS | 1 |
| 98 | LiC－ PL－AS－ HP → RG | 1 |
| 99 | PL－ RG－ HP → LiC－ AS | 0.98 |
| 100 | PL－ AS－ HP → LiC－ RG | 0.98 |
| 101 | LiC－ PL－ HP → AS－ RG | 1 |
| 102 | PL－ HP → LiC－ AS－ RG | 0.98 |
| 103 | LyC－AS－ RG－ HP → LiC | 1 |
| 104 | LiC－ LyC－ RG－ HP → AS | 1 |
| 105 | LiC－ LyC－AS－ HP → RG | 0.96 |
| 106 | LyC－ RG－ HP → LiC－AS | 1 |
| 107 | LyC－ AS－ HP → LiC－ RG | 0.96 |


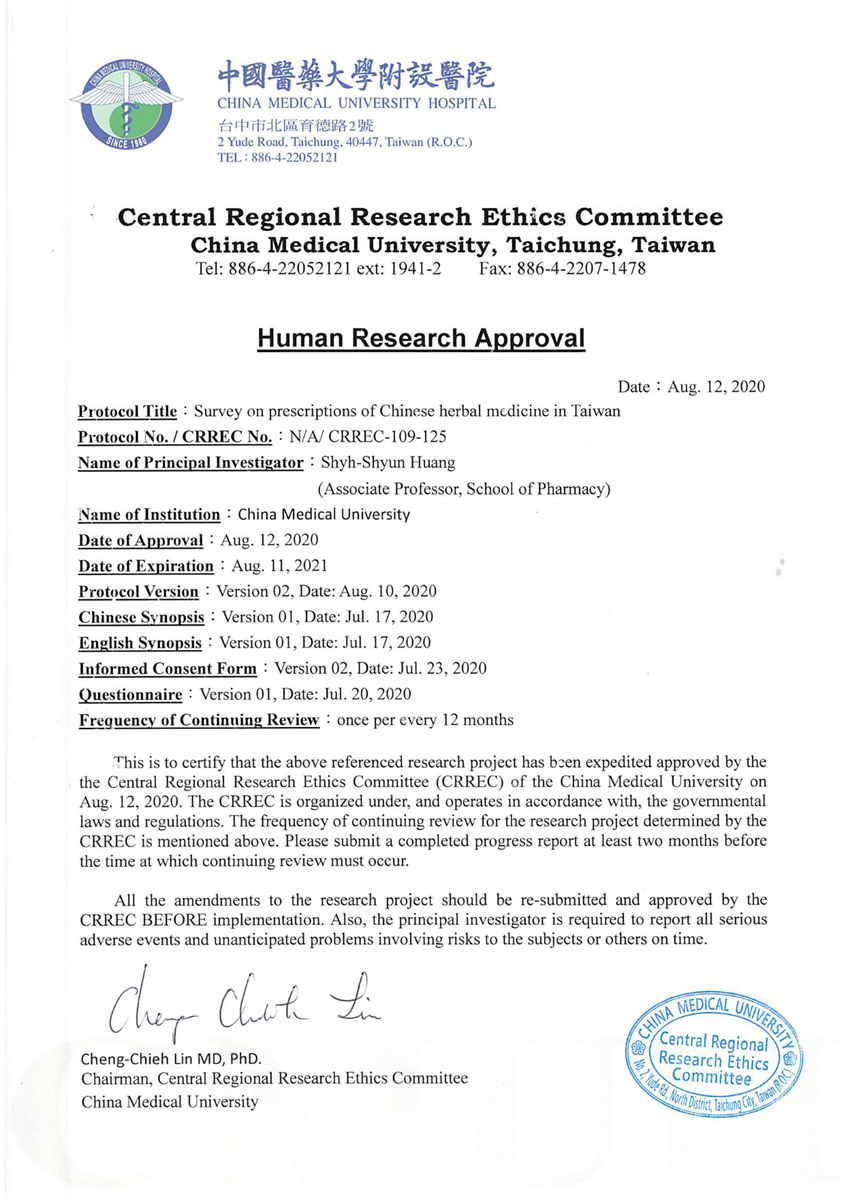


**Figure S1.** Approval letter for the ethnopharmacological survey of traditional Chinese medicine pharmacies in Taiwan issued by the Central Regional Research Ethics Center of China Medical University

| 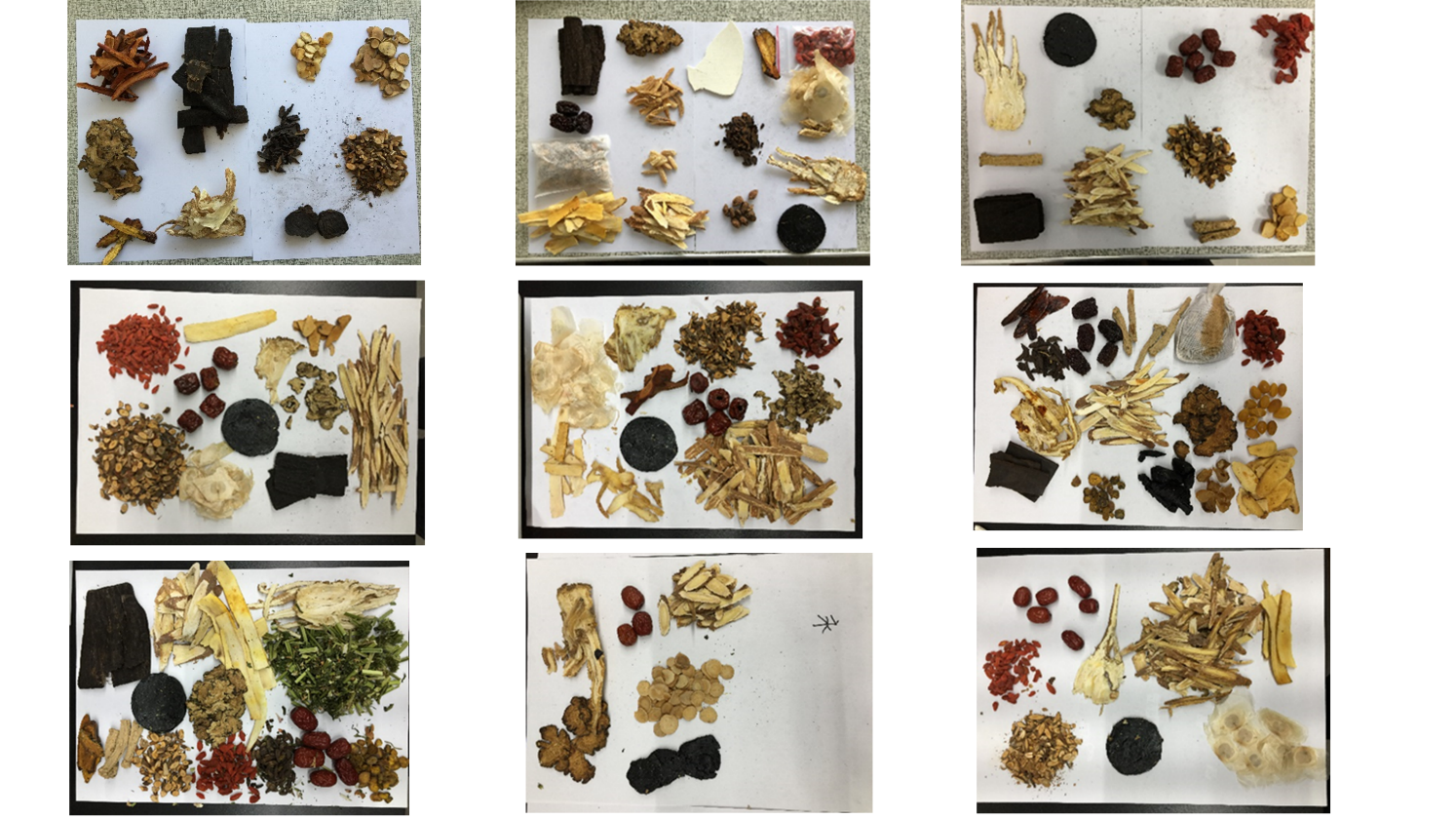 |
| --- |
|  |
| 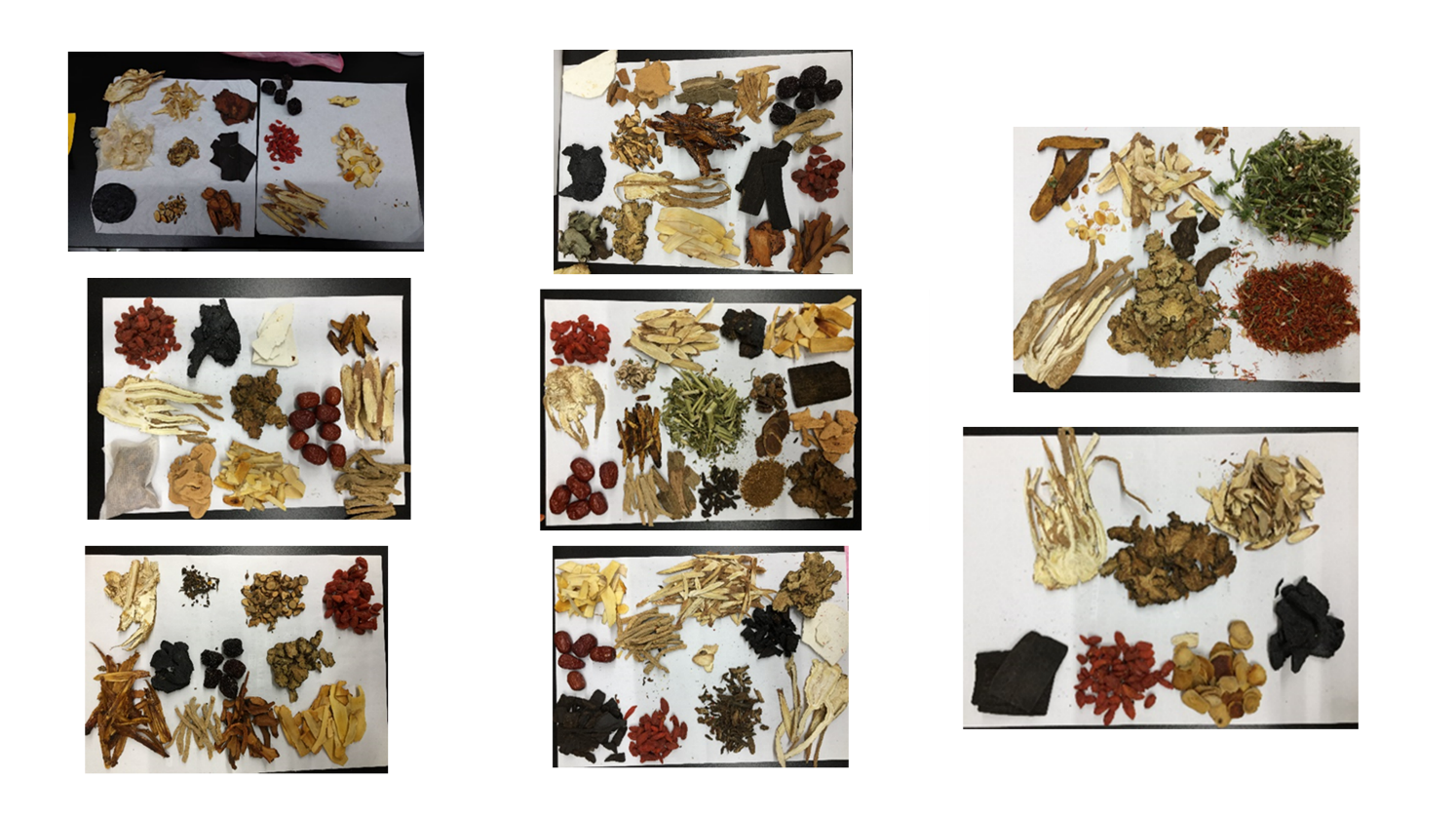 |
| 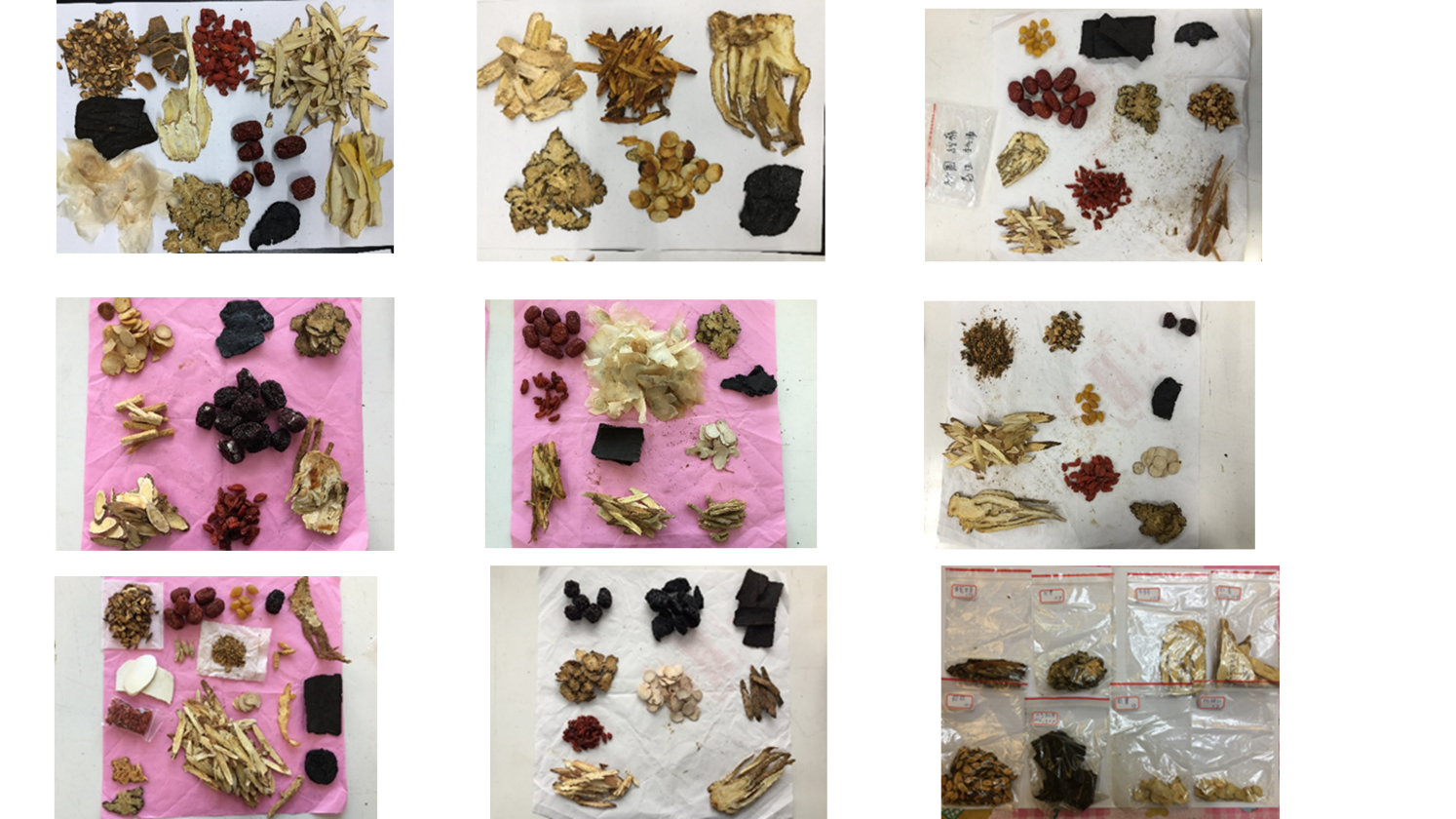 |
|  |
| 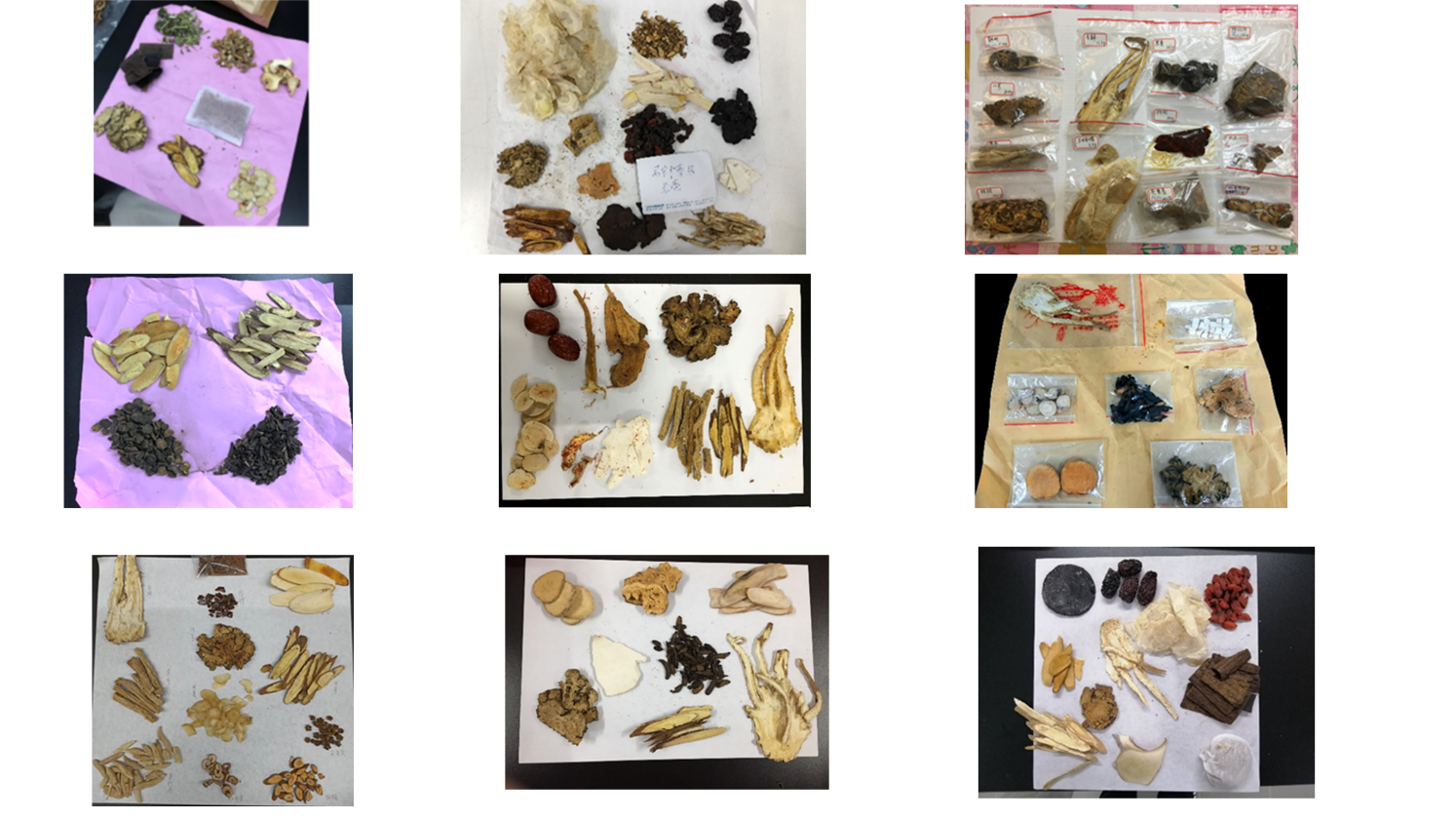 |
| 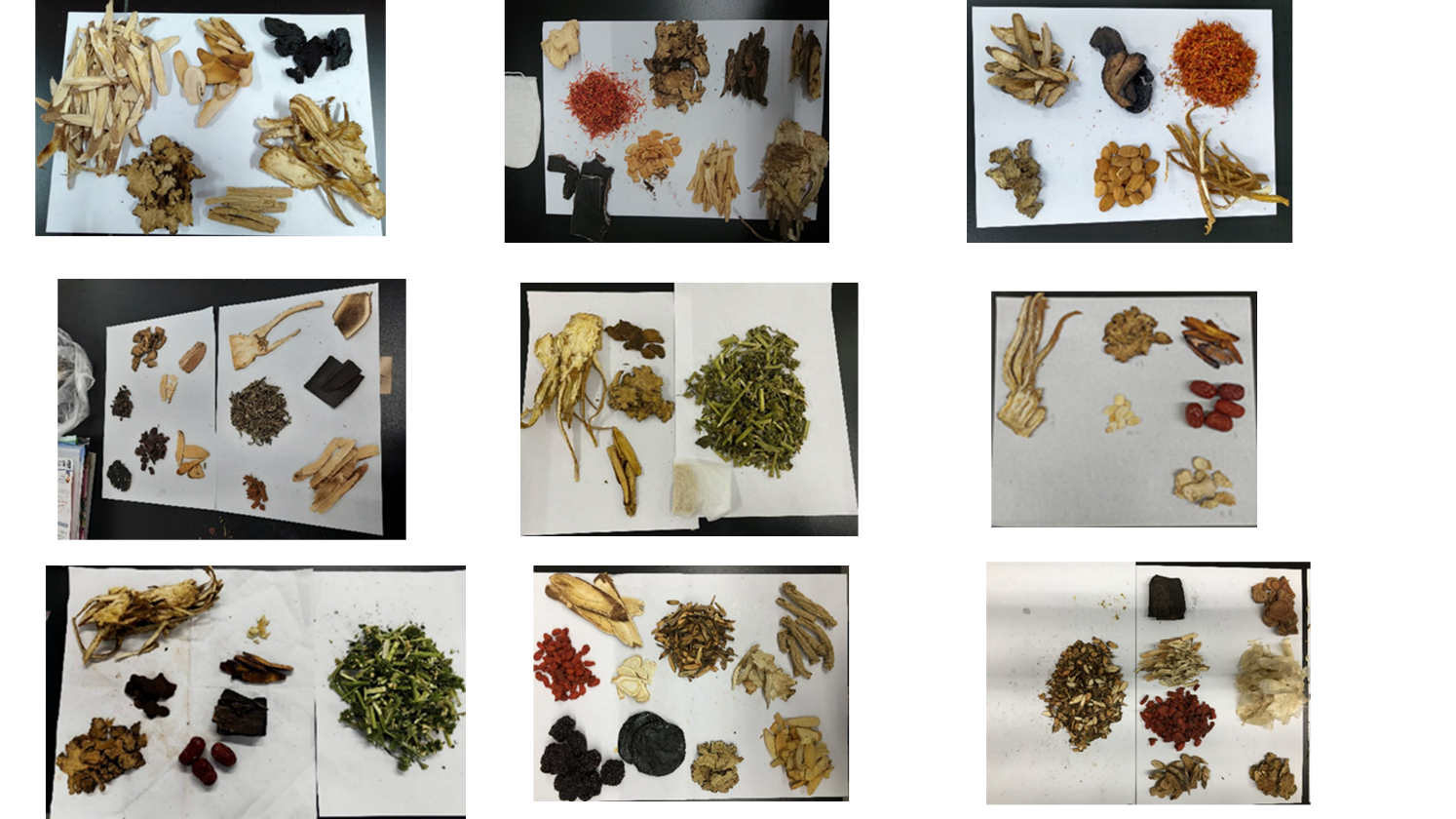 |
|  |
| 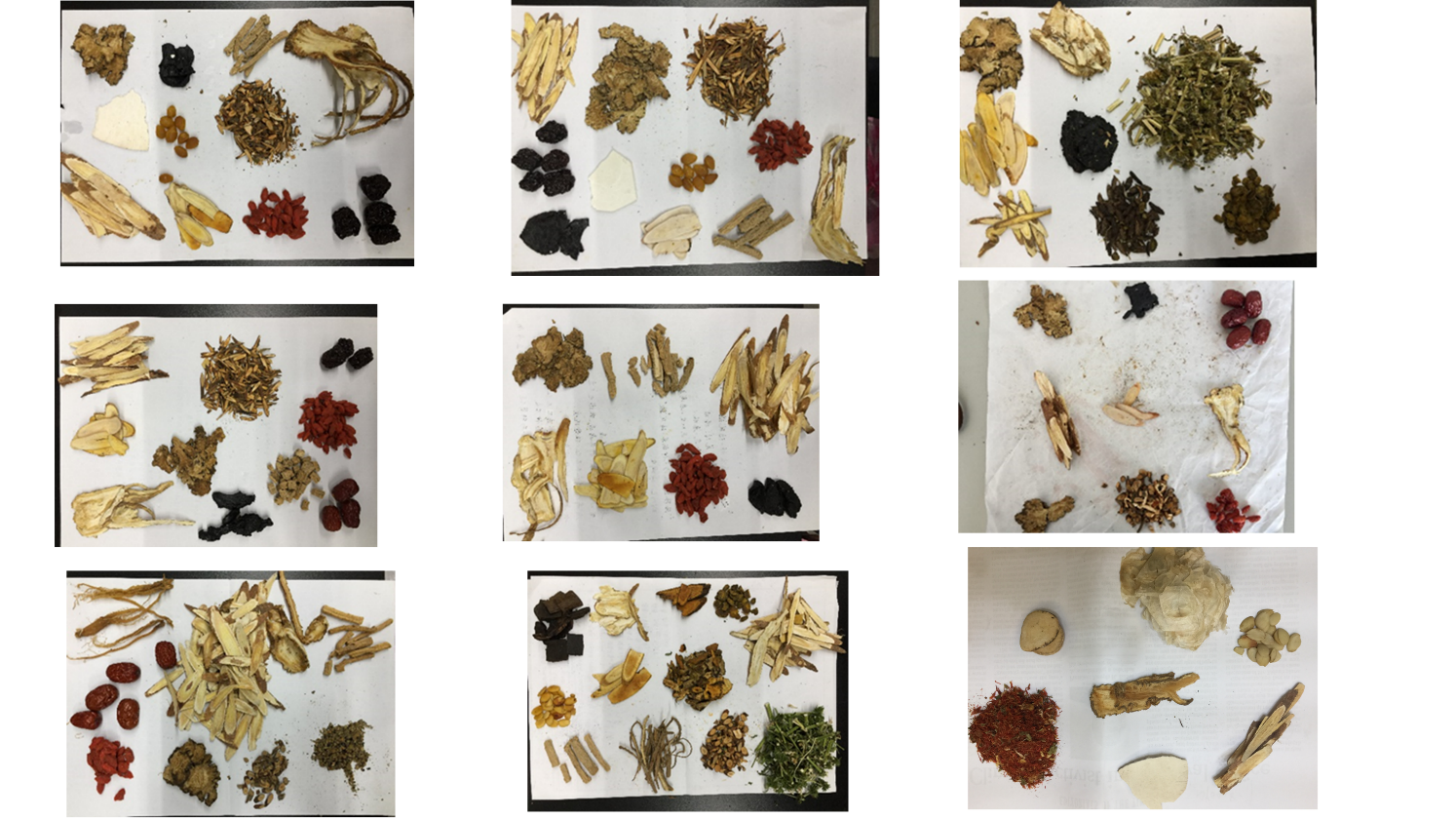 |
| 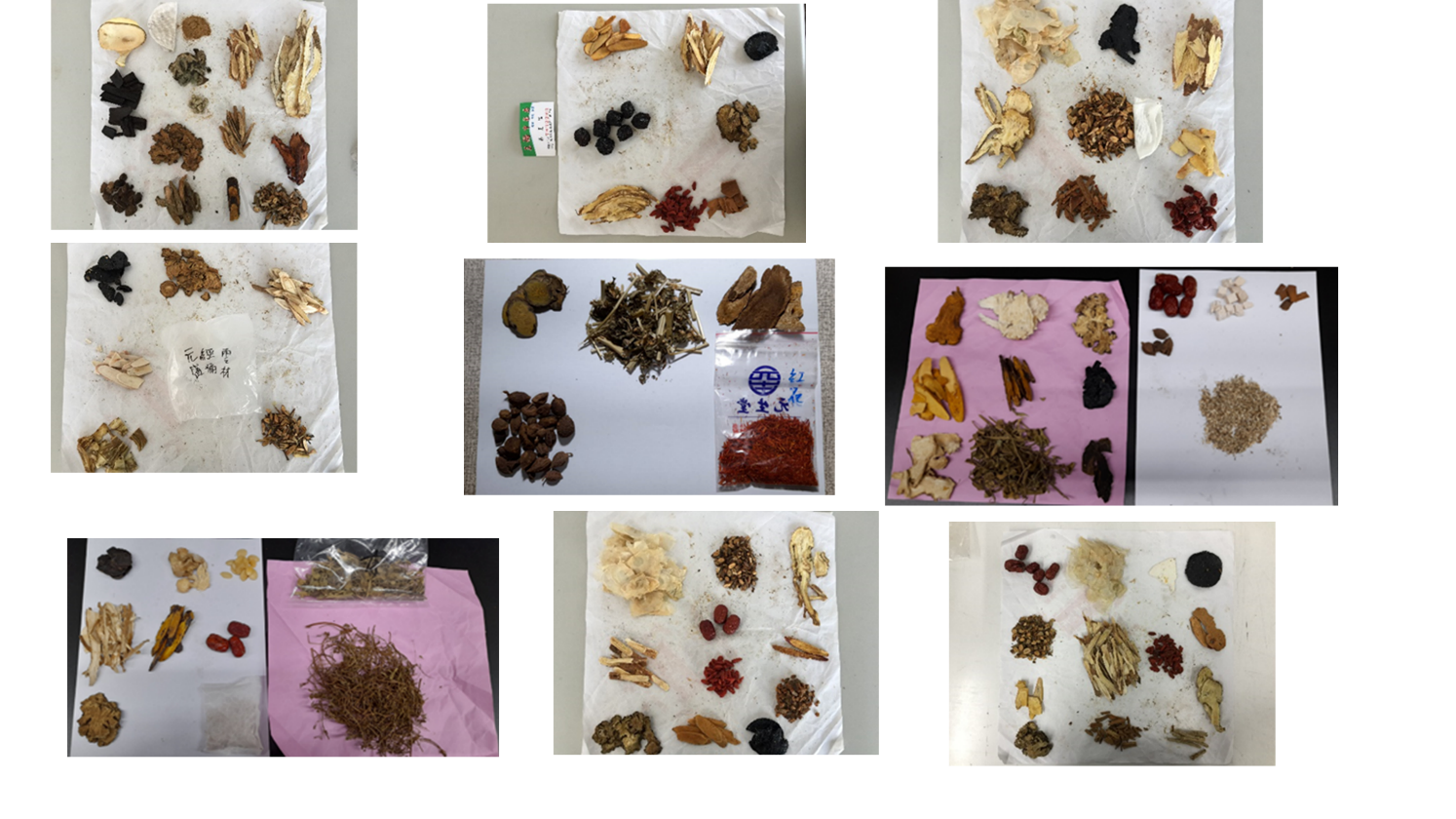 |
|  |
| 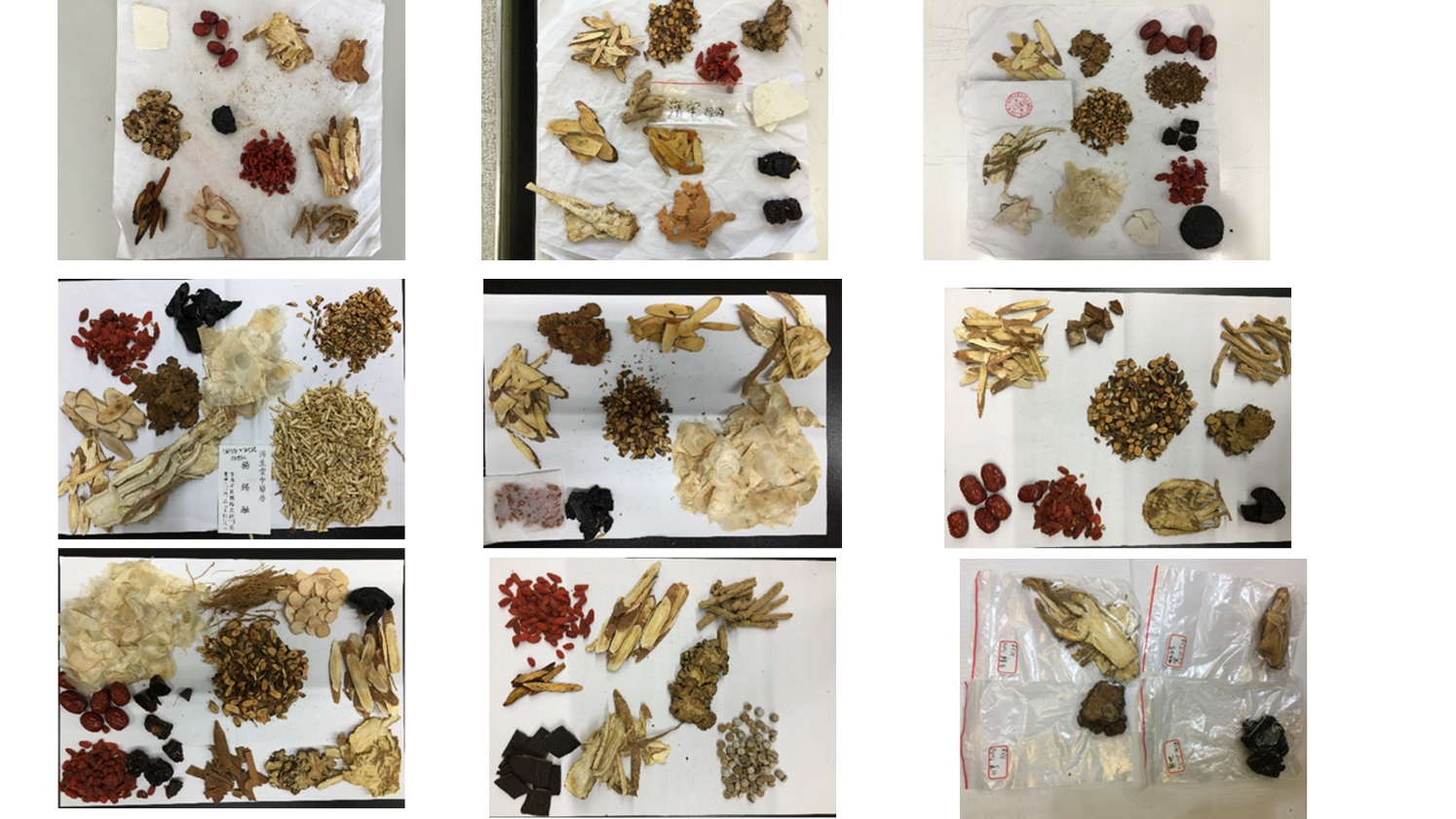 |
| 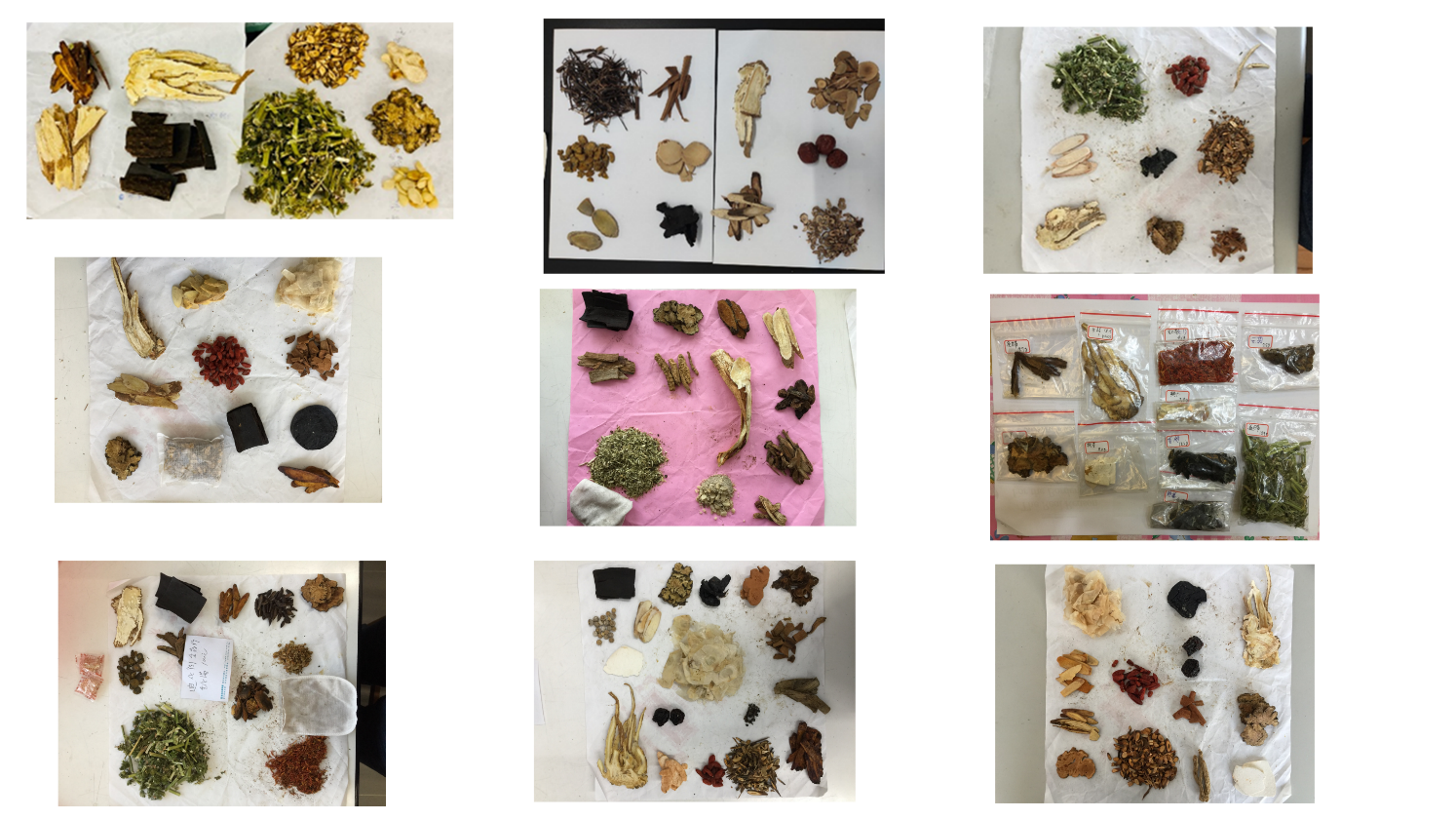 |
|  |
| 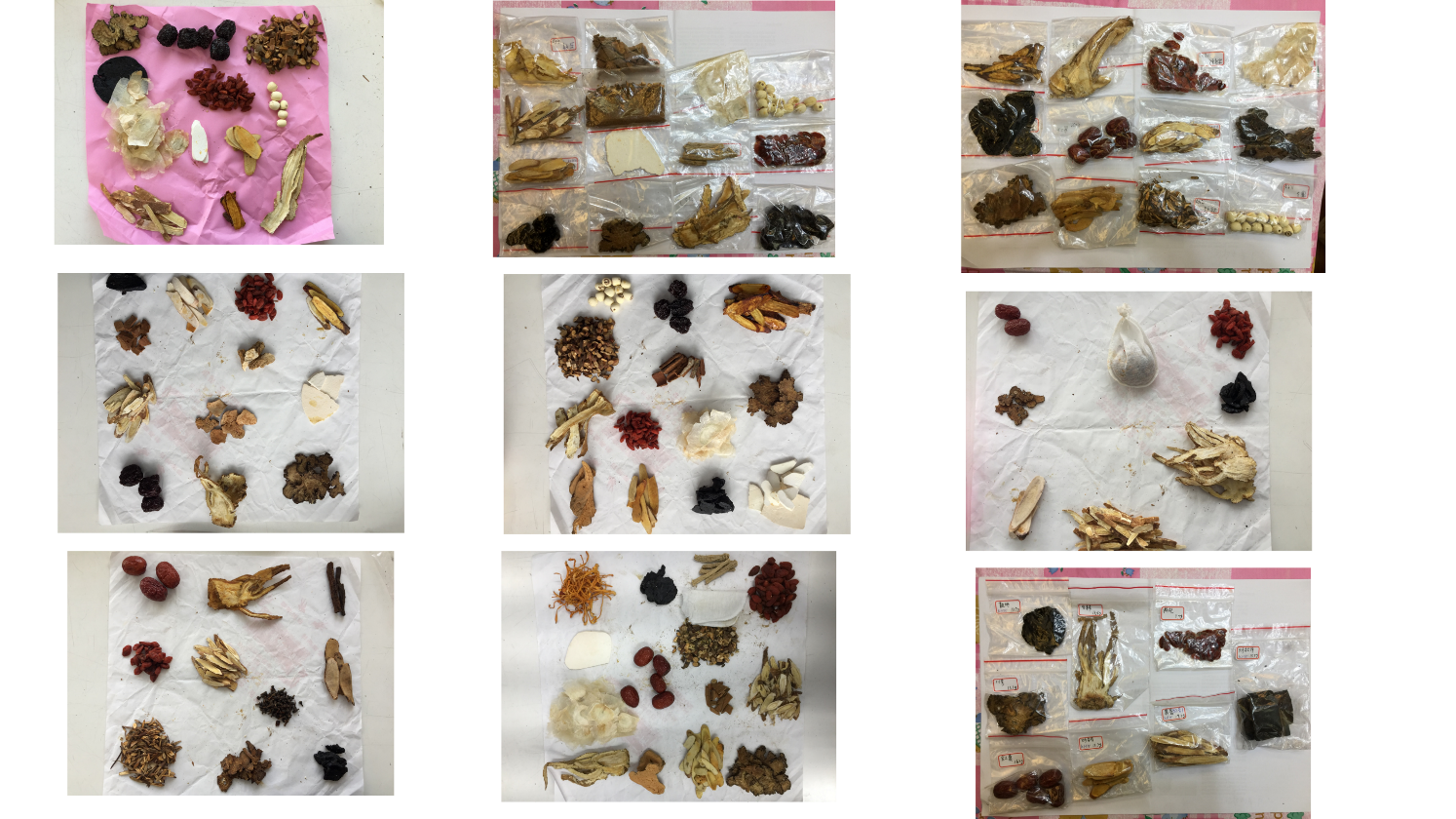 |
| 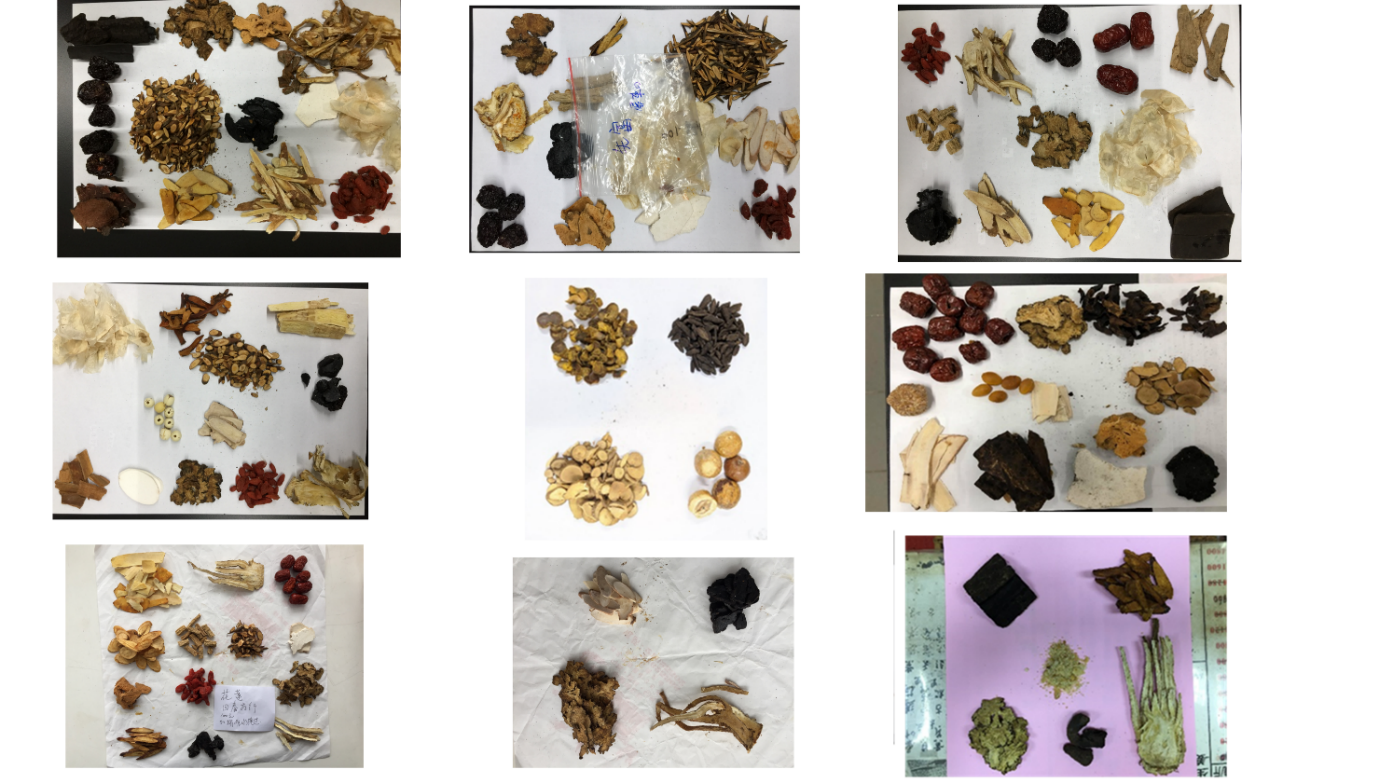 |

**Figure S2.** Photographs of the 99 prescriptions for dysmenorrhea collected in this study
